# Robust walking control of a lower limb rehabilitation exoskeleton coupled with a musculoskeletal model via deep reinforcement learning

**DOI:** 10.1186/s12984-023-01147-2

**Published:** 2023-03-19

**Authors:** Shuzhen Luo, Ghaith Androwis, Sergei Adamovich, Erick Nunez, Hao Su, Xianlian Zhou

**Affiliations:** 1 Department of Biomedical Engineering, New Jersey Institute of Technology, Newark, 07102 NJ USA; 2 Kessler Foundation, West Orange, 07052 NJ USA; 3 Lab of Biomechatronics and Intelligent Robotics, Department of Mechanical and Aerospace Engineering, North Carolina State University, Raleigh, 27695 NC USA; 4 Joint NCSU/UNC Department of Biomedical Engineering, University of North Carolina at Chapel Hill, Chapel Hill, 27599 NC USA

**Keywords:** Robust walking control, Human-exoskeleton interactions, Muscular disorders, Deep reinforcement learning

## Abstract

**Background:**

Few studies have systematically investigated robust controllers for lower limb rehabilitation exoskeletons (LLREs) that can safely and effectively assist users with a variety of neuromuscular disorders to walk with full autonomy. One of the key challenges for developing such a robust controller is to handle different degrees of uncertain human-exoskeleton interaction forces from the patients. Consequently, conventional walking controllers either are patient-condition specific or involve tuning of many control parameters, which could behave unreliably and even fail to maintain balance.

**Methods:**

We present a novel, deep neural network, reinforcement learning-based robust controller for a LLRE based on a decoupled offline human-exoskeleton simulation training with three independent networks, which aims to provide reliable walking assistance against various and uncertain human-exoskeleton interaction forces. The exoskeleton controller is driven by a neural network control policy that acts on a stream of the LLRE’s proprioceptive signals, including joint kinematic states, and subsequently predicts real-time position control targets for the actuated joints. To handle uncertain human interaction forces, the control policy is trained intentionally with an integrated human musculoskeletal model and realistic human-exoskeleton interaction forces. Two other neural networks are connected with the control policy network to predict the interaction forces and muscle coordination. To further increase the robustness of the control policy to different human conditions, we employ domain randomization during training that includes not only randomization of exoskeleton dynamics properties but, more importantly, randomization of human muscle strength to simulate the variability of the patient’s disability. Through this decoupled deep reinforcement learning framework, the trained controller of LLREs is able to provide reliable walking assistance to patients with different degrees of neuromuscular disorders without any control parameter tuning.

**Results and conclusion:**

A universal, RL-based walking controller is trained and virtually tested on a LLRE system to verify its effectiveness and robustness in assisting users with different disabilities such as passive muscles (quadriplegic), muscle weakness, or hemiplegic conditions without any control parameter tuning. Analysis of the RMSE for joint tracking, CoP-based stability, and gait symmetry shows the effectiveness of the controller. An ablation study also demonstrates the strong robustness of the control policy under large exoskeleton dynamic property ranges and various human-exoskeleton interaction forces. The decoupled network structure allows us to isolate the LLRE control policy network for testing and sim-to-real transfer since it uses only proprioception information of the LLRE (joint sensory state) as the input. Furthermore, the controller is shown to be able to handle different patient conditions without the need for patient-specific control parameter tuning.

**Supplementary Information:**

The online version contains supplementary material available at 10.1186/s12984-023-01147-2.

## Introduction

Wearable robots like lower-limb exoskeletons have great potential for mobility restoration and human augmentation [1]. Scientific and technological work on exoskeletons began in the early 1960s but have only been recently applied for gait assistance rehabilitation and functional substitution in patients suffering from motor disorders. There are two main types of exoskeletons for gait assistance: the ones for partial assistance and the others for full mobilization. Partial assistance exoskeletons are generally lighter, targeting less severe handicaps. They can also assist healthy people for performance or endurance augmentation purposes [2]. Full mobilization exoskeletons are designed to move the legs of people suffering from severe loss of motor control or motor disorders, typically in people with spinal cord injury (SCI) and neuromuscular disorders [3, 4], to perform activities of daily living (ADL) [2, 5–8]. Lower limb rehabilitation exoskeletons (LLREs) with multi-joint actuation for full mobilization have been used more often nowadays in rehabilitation clinics and have shown great benefits to improve mobility for people with a variety of neuromuscular disorders such as muscle weakness or paralysis) [3, 4, 9, 10]. Further investigation on LLREs to assist people with neuromuscular disorders is an important part of rehabilitation exoskeleton (RE) research frontiers [9, 10].

Robustness and stability of the LLRE for walking assistance is of great significance to ensure the safety of the patient. One of the most common ways to ensure that is to use crutches or other balance assistance devices for additional support to avoid falling down during walking. Some commercially available exoskeletons include ReWalk (ReWalk Robotics), Ekso (Ekso bionics), Indego (Parker Hannifin), TWIICE [6], VariLeg [11] and LFMAS [12]. ReWalk measures the tilt angle of the upper body to initiate walking, and Ekso uses accelerometers on crutches and pressure sensors on shoes to detect the walking intention of the wearer. However, holding the crutches with the arms and hands limits the patient’s interactions with the environment [13] and hinders the patient’s timely response to emergencies. In addition, it adds additional burden to the patient’s upper body. A limited number of LLREs are able to assist human walking without the need of crutches or helpers, such as Rex (Rex Bionics) [14] and Atalante (Wandercraft) [15]. These LLREs free the user’s hands, but come at the cost of very low walking speeds and increased overall weights (38 kg for the Rex and 60 kg for the Atalante). In addition, these heavy autonomous LLREs are very expensive [6]. In this paper, we target the robust control of a lightweight LLRE currently being developed in our group [16, 17] that includes a sufficient number of degrees of freedom (DoF) and has very strong actuation. The goal is to enable autonomous, independent walking with this LLRE without external help, which could give the patient a confidence boost to use the LLRE in the clinical or home setting. In order for it to cooperate with human with minimal risks of fall or physical harm, advanced controllers to robustly perform walking assistance under various human-exoskeleton interaction conditions need to be developed.

There are many challenges in developing such advanced controllers due to inherent requirements of safe interaction with the patient and the environment [2, 18, 19]. Because of varied conditions of patients’ disabilities, the human-exoskeleton interaction forces are unpredictable and could vary substantially from one patient to another, a very important factor to consider for controller development. Existing controllers for LLREs often focus on trajectory tracking, conventional Proportional-Integral-Derivative (PID) control [20], fuzzy control [8], model-based predictive control [21], impedance control [22, 23], and momentum-based control [24]. The trajectory tracking approaches are primarily used for early-stage rehabilitation when patients have very weak muscle strength, its robustness against unexpected large perturbations or uncertain interaction forces is not great. Model-based method could be ineffective or even unstable due to inaccurate dynamics modeling, and it typically requires a laborious task-specific control parameter tuning. To overcome the model uncertainties, data-driven, RL-based controllers are attracting attention in the LLRE control recently [12, 25–27]. In [26], a human-exoskeleton interaction control of a gait rehabilitation LLRE was proposed and the proposed adaptive law of the admittance parameters was designed with the RL algorithm. This kind of tethered rehabilitation robot is less portable and can be used only in laboratory and clinical applications. In our previous work [17], a reinforcement learning-based controller for a LLRE squatting motion control was developed through training with a tightly coupled human-exoskeleton simulation, for which the input to the neural network-based controller includes the human full body state, the exoskeleton proprioceptive information, and the foot Center of Pressure (CoP) positions. The requirement on all these sensory inputs (especially the human state) makes it difficult to transfer the trained controller to the LLRE hardware.

This paper is the first investigation of a deep neural network-based reinforcement learning (RL) controller for LLREs to realize robust walking control without any control parameter tuning. The method proposed in this work offers several distinct advantages: (1) This controller is trained from the decoupled offline human-exoskeleton simulation; this decoupled simulation structure enables the trained control policy to use only proprioceptive information (encoder data) of the LLRE regardless of the uncertain human-exoskeleton interaction forces from different levels of muscle weakness, which consequently facilitates easy deployment of the controller to the physical exoskeleton. (2) We employ domain randomization during training that includes not only randomization of exoskeleton mechanical properties but, more importantly, randomization of human muscle strength to simulate the variability of the patient’s disability, in order to produce a universal controller without any control parameter tuning. Learning with muscle strength randomization allows the RL controller of the exoskeleton to produce a universal, self-adaptive walking control policy to handle varying human-exoskeleton interactions from subjects with different degrees of neuromuscular disability without any manual tuning of control parameters. The proposed decoupled RL-based walking control strategy is virtually tested on a LLRE system to verify its effectiveness in assisting users with different disabilities such as passive muscles (quadriplegic), muscle weakness, or even hemiplegic conditions. The RMSE for joint tracking accuracy, CoP-based stability, and symmetry analysis show the effectiveness of the controller. An ablation study also demonstrates the strong robustness of the control policy under large exoskeleton dynamic property ranges and various human-exoskeleton interaction forces.

## Exoskeleton and interaction modeling

### Modeling of a LLRE

**Fig. 1:**
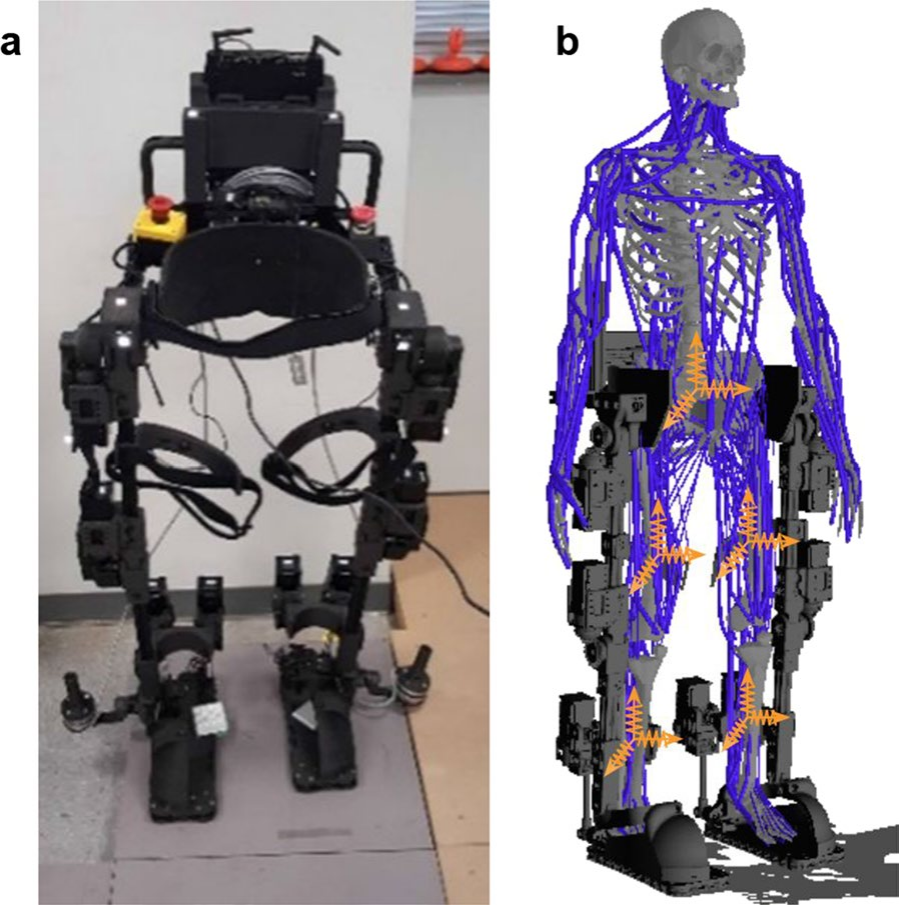
The integrated human and exoskeleton model. **a** The physical prototype of the LLRE. **b** The integrated musculoskeletal and exoskeleton model. The yellow coordination frames show the bushing frames coincidentally fixed on the LLRE and the human

A LLRE hardware shown in Fig. 1a has been developed in an early effort [16] to assist patients with gait rehabilitation. The details about the LLRE design have been presented in our prior work [17]. This LLRE system has 8 actuated DoFs, each side of the body includes 1 DoF for the hip flexion/extension, 1 DoF for the knee flexion/extension, and 2 DoFs for the ankle. The LLRE uses smart servo motors (Dynamixel Pro H54–200-S500-R) for hip, knee, and ankle joints. Both hip and knee joints are driven by bevel gears with a 3:1 gear ratio for compact design and are able to provide a continuous torque (under a continuous 9.3 A current) and speed of 132 Nm and $55^\circ /s$, respectively. The motor’s max current is close to 14*A* at which it can generate over 220 Nm torque with the 3:1 supplemental gear. The range of motion of the hip is from ${-80^{\circ }}$ (extension) to ${80^{\circ }}$ (flexion) and the knee joint has a range from ${0^{\circ }}$ (straight knee) to $160^{\circ }$ (flexion). In contrast to most commercial LLREs with passive or fixed ankles, the ankle of our system consists of a powered 2-DoF joint to assist with dorsiflexion/plantarflexion and inversion/eversion with torque over 160 Nm [28]. These 2 DoFs have their rotation axes located at different positions and are physically driven by the closed-loop of two ankle motors together with linkage of universal joints and screw joints. Besides these 8 actuated DoFs, the root joint in the model of the LLRE system has 6 unactuated DoFs (3 global transnational and 3 global rotational DoFs) to allow its free movement in space. The total mass of the exoskeleton is 20.4 kg and the frame of the exoskeleton has been manufactured with Onyx (Markforged’s nylon with chopped fiber) reinforced by continuous carbon fiber between layers, using Markforged’s Mark Two printer (Markforged, INC., MA). A 24V high capacity rechargeable Lithium Ion battery (Bixpower CP330-BX2499) is used to power the LLRE.

### Modeling of human exoskeleton interactions

#### Musculoskeletal modeling

To simulate realistic human-exoskeleton interaction, a full-body human musculoskeletal model used in [29] is integrated with the LLRE to create realistic human-exoskeleton interaction forces and constraints. The musculoskeletal model, illustrated in Fig. 1, is around 170 cm tall, weighs 72 kg and consists of 50 DoFs and 284 musculotendon units. Each musculotendon unit is represented as a polyline that starts at the origin of the muscle, passes through a sequence of waypoints, and ends at the insertion. It generates an active muscle force through contraction and applies the force to the two bones of its origin and insertion. The contraction muscle dynamics is simulated with a simplified Hill-type model [30, 31] as follows,

1 $$\begin{aligned} F= [a \cdot F_L(l) \cdot F_V(\dot{l}) +F_P(l)]\times F_{max} \end{aligned}$$

where $a \in [0, 1]$ is the muscle activation, $F_{max}$ is the maximum isometric muscle force, and *l* is the normalized muscle length. $F_L$ and $F_V$ are force-length and force-velocity functions, respectively. When the muscle is fully passive without active contraction ($a = 0$), it develops only a passive force $F_P \times F_{max}$ because of its background elasticity. The Euler–Lagrangian equations for the human musculoskeletal dynamics using generalized coordinates can be described by:

2 $$\begin{aligned} \varvec{M}(\varvec{q})\ddot{\varvec{q}} +\varvec{c}(\varvec{q},\dot{\varvec{q}}) = \varvec{J}_m^T\varvec{f}_m(\varvec{a}) + \varvec{J}_{ext}^T\varvec{f}_{ext} \end{aligned}$$

where $\varvec{q}$ is the vector of joint angles, $\varvec{f}_{ext}$ is the vector of external forces, and $\varvec{f}_m$ is the vector of muscle forces which is a function of muscle activations $\varvec{a}=(a_1,a_2, \cdots , a_n)$ for all muscles. $\varvec{M}(\varvec{q})$ denotes the generalized mass matrix, and $\varvec{c}(\varvec{q},\dot{\varvec{q}})$ is Coriolis and gravitational forces. $\varvec{J}_m$ and $\varvec{J}_{ext}$ are Jacobian matrices that map the muscle and external forces to the joint space, respectively. Following [29], due to the linearity of muscle force over the activation, we can write the muscle force vector

3 $$\begin{aligned} \varvec{f}_m(\varvec{a}) = \frac{\partial \varvec{f}_m}{\partial \varvec{a}} \varvec{a} +\varvec{f}_m(\varvec{0}). \end{aligned}$$

Consequently, Eq. 2 can be rewritten as

4 $$\begin{aligned} \varvec{M}(\varvec{q})\ddot{\varvec{q}} +\varvec{c}(\varvec{q},\dot{\varvec{q}}) = \varvec{A}\varvec{a} + \varvec{e} + \varvec{J}_{ext}^T\varvec{f}_{ext} \end{aligned}$$

with

5 $$\begin{aligned} \varvec{A} =\varvec{J}_m^T\frac{\partial \varvec{f}_m}{\partial \varvec{a}}, ~~\varvec{e} = \varvec{J}_m^T\varvec{f}_m(\varvec{0}). \end{aligned}$$

As a result, the joint coordinate acceleration $\varvec{\ddot{q}}$ can be computed from

6 $$\begin{aligned} \ddot{\varvec{q}} =\varvec{Ka+b} \end{aligned}$$

with

7 $$\begin{aligned} \varvec{K=M^{-1}A, ~~b=M^{-1}(e+\varvec{J}_{ext}^Tf_{ext}-c)}. \end{aligned}$$

#### Modeling of human-exoskeleton interactions

The LLRE has straps around the hip, femur, and tibia to constrain human motion, as shown in Fig. 1a. In this study, the pelvis of the human musculoskeletal model is attached to the exoskeleton hip through a prismatic joint that allows relative movement only along the vertical (up and down) direction. Meanwhile, we use linear bushing elements [32] to simulate the interaction forces and moments between the human and exoskeleton at all strap locations. A linear bushing element represents a bushing connecting a frame fixed on the exoskeleton to a frame fixed on the human with linear translational and torsional springs and dampers. The yellow coordination frames in Fig. 1b show the bushing frames coincidentally fixed on the LLRE and the human during initial alignment. The governing equations for the bushing element are as follows:

8 $$\begin{aligned} \left\{ \begin{array}{l} f_{x} = k_{x} x + c_{x} {\dot{x}} \\ f_{y} = k_{y} y + c_{y} {\dot{y}} \\ f_{z} = k_{z} z + c_{z} {\dot{z}} \\ \end{array}\right. , and \left\{ \begin{array}{l} \tau _{x} = \alpha _{x} \theta _{x} + \beta _{x} {\dot{\theta }}_{x} \\ \tau _{y} = \alpha _{y} \theta _{y} + \beta _{y} {\dot{\theta }}_{y} \\ \tau _{z} = \alpha _{z} \theta _{z} + \beta _{z} {\dot{\theta }}_{z} \\ \end{array}\right. \end{aligned}$$

where $f_x$, $f_y$ and $f_z$, are the translational forces; $\tau _x$, $\tau _y$, and $\tau _z$ are the rotational or torsional moments along the bushing frames; *x*, *y*, *z* are the translation distances between the origins of the two frames; $\theta _x$, $\theta _y$, and $\theta _z$ are the $x-y-z$ body fixed Euler angles between the frames; $k_i$, $c_i$, $\alpha _i$, and $\beta _i$ ($i=x,y,z$) denote directional linear constants. These directional constants allow us to model different resistance strengths of straps along different directions. During motion, bushing forces and moments are generated due to deviation of the two frames and they are applied to both human and exoskeleton. For the interaction between the human pelvis and the LLRE waist structure, the bushing element only generates a force along the vertical direction and we specify its translation stiffness $k_y=8000$, translation damping $c_y = 10$. At the other four leg strap locations, the following bushings parameters are used: translation stiffness $k_x =k_z=1500, k_y=500$, translation damping $c_x=c_z=10,c_y=1$, rotation stiffness $\alpha _x= \alpha _z =10, \alpha _y =3$, rotation damping $\beta _x =\beta _z= 1, \beta _y=0.1$ to simulate the connections between the human femur and the strap on the LLRE femur. We use smaller constants along the limb length (axis) direction to allow the straps to slide up and down and rotate along the axis direction easier. Besides using these bushing elements for elastic strap modeling, we assume there is no relative motion between the human foot and the exoskeleton foot due to tight coupling and model that as a fixed constraint.

**Fig. 2:**
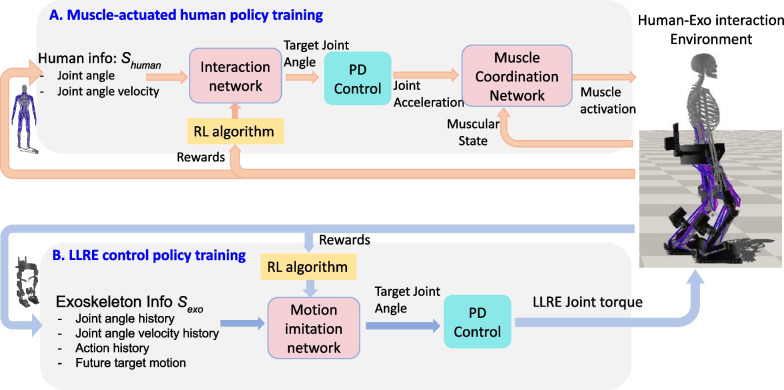
Overview of the modular, decoupled RL-based walking control framework of the LLRE with human-in-the-loop. The framework is separated into two parts: **A** muscle-actuated human policy training. **B** LLRE control policy training, which integrates three deep neural networks (marked with pink blocks): an RL-based interaction network for human control to manage human-exoskeleton interface forces; a supervised muscle coordination network for whole body muscle control; a RL-based motion imitation network for the control of the LLRE. The muscle-actuated human control loop **A** is designed to learn human muscle coordination giving the health status of the human and predicted exoskeleton assistance. The LLRE control loop **B** is designed to imitate a target walking motion while maintaining strong robustness and balance under the human-exoskeleton interaction. These three networks can be jointly trained in the simulation while they interact with each other to achieve maximum rewards during RL

## Deep RL-based walking control

In this section, we present a novel walking controller based on deep RL with decoupled neural networks for the LLRE to perform walking assistance with strong robustness against various human-exoskeleton interactions. Figure 2 shows the overall learning framework of this deep RL-based walking control workflow, it includes (A) a muscle-actuated human control loop and (B) a LLRE control loop. The goal of the LLRE control loop is to learn a control policy $\pi _{\theta }(a_s|s_{exo})$ of the LLRE that imitates a target walking motion while achieving strong robustness and balance under the influence of the human-exoskeleton interaction. The motion imitation network (pink block) in this loop is a stochastic control policy $\pi _{\theta }(a_s|s_{exo})$ of the LLRE. The human muscle-actuated control loop is required to generate realistic human-exoskeleton interactions. In the muscle-actuated control loop, a combination of an RL-based interaction network and a supervised learning-based muscle coordination network (pink blocks) is devised to learn human muscle activation during assisted walking. The first RL-based interaction network aims to produce small interaction forces between the human and the LLRE by considering the patient’s desire to follow the exoskeleton movement and reduce pressure on the body. This interaction network takes the human skeleton state $s_{human}$ (the kinematic states of the human) as the input and its policy $\pi _{\phi }(a_h|s_{human})$ produces target human joint angle output ($a_h$) during human-exoskeleton interaction, where $\phi$ is network parameters to be optimized using RL. PD control from these target angles generates desired human joint accelerations $q_d$, which are passed to the second muscle coordination network. The muscle coordination network $a_m = \pi _{\varphi } (q_d, s_{muscle})$ is a deterministic policy that outputs the muscle activations $a_m$ from the current muscle state $s_{muscle}$ to minimize the differences between the muscle generated acceleration and the desired joint acceleration, where $\varphi$ is network parameters determined by regression. Collectively, these three networks are jointly learned through simulations to achieve maximum rewards in deep RL. Through this proposed decoupled learning control process, the controller for the LLRE will be shown to be able to handle varying human-exoskeleton interactions caused by different degrees of human disability just using proprioception information of the LLRE (joint sensory state). In the following subsections, the details of these three control networks are introduced.

### RL-based LLRE control policy training

**Fig. 3:**
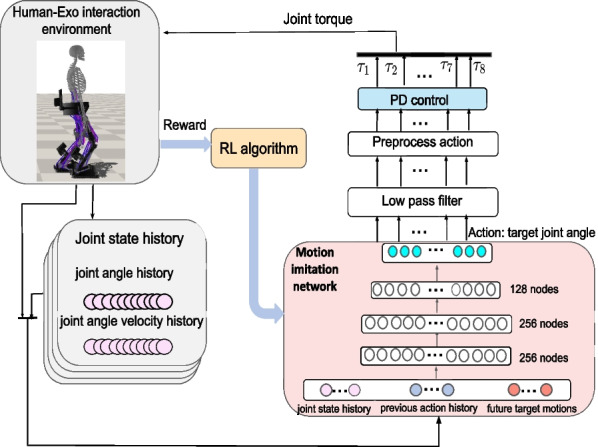
RL-based motion imitation control of the LLRE (the LLRE control loop in Fig. 2). The inputs of the motion imitation network consist of the joint state history, the action history and the future target motions. This learning network outputs joint target positions, which are processed by a low-pass filter and then translated into torque-level commands by PD control

Figure 3 shows a detailed schematic of the RL-based LLRE control loop (B in Fig. 2). The controller (or control policy) is learned through a continuous RL process. We design the control policy through a neural network with parameters $\theta$, denoting the weights and bias in the neural network. The control policy can be expressed as $\pi _{\theta }(a_s|s_{exo})$ and the parameters $\theta$ of the neural network are updated according to the policy gradient method to achieve the maximum reward. In the learning process (Fig. 3), the input of the control policy network is defined by $s_{exo}=\{p_{t-2:t},v_{t-2:t},a_{t-2:t},{\hat{p}}_{t+1:t+6}\}$, in which *p* and *v* are joint angles and angular velocities of the LLRE, and $a_{t-2:t}$ represents the action history of three sequential steps. To learn a particular skill, we utilize the corresponding target joint poses from the task motion at six future time-steps ${\hat{p}}_{t+1:t+6}$ as the motion prior for feasible control strategies. The use of task motion data, despite being task-specific, alleviates the need to design task-specific reward functions and thereby facilitates a general framework to learn a diverse array of behaviors.

In Fig. 3, the control policy network is implemented as a Multi-Layer Perception (MLP) network that consists of three fully connected layers and ReLU as the activation function. The sizes of three layers are set to 256, 256 and 128, respectively. At every time step *t*, the neural network model observes the state of the exoskeleton $s_{exo,t}$ from the environment, and selects an action $a_{s,t}$ according to its control policy $\pi _\theta (a_{s}|s_{exo})$. $\pi _\theta (a_{s}|s_{exo})$ is in the form of the probability distribution of actions in a given state. The LLRE in the environment then applies the action $a_{s,t}$, which results in a new state $s_{exo,t+1}$ and a scalar reward $r_t$ immediately. The objective is to learn a control policy that maximizes the discounted sum of reward:

9 $$\begin{aligned} \pi ^*= \mathop {\arg \max }_{\pi } \mathbb {E}_{\tau \sim p(\tau |\pi )}\left[ \ \sum _{t=0}^{T-1}\gamma ^tr_t\right] \ \end{aligned}$$

where $\gamma \in (0,1)$ is the discount factor, $\tau$ is the trajectory $\{(s_0,a_0,r_0),(s_1,a_1,r_1), \cdots \}$ and $p(\tau |\pi )$ denotes the likelihood of a trajectory $\tau$ under a given control policy $\pi$. *T* is the horizon of an episode. We design the reward function $r_t = w^pr_p+ w^er_e+w^{root}r_{root}+ w^{cop}r_{cop}+ w^{\tau }r_{\tau }+w^{as}r_{as} + w^{fc}r_{fc}$ as the weighted summation of multiple sub-rewards to encourage the control policy to imitate a target walking motion while maintaining balance with robustness. *w* denotes the corresponding weight for each sub-reward. The list of sub-rewards is itemized as follows:

- Imitation Reward ($r_p$ and $r_e$): These two terms encourage the exoskeleton to minimize the difference between the current and reference motions in terms of the joint positions ($p_t$) and end-effector positions ($x_t$).
  10 $$\begin{aligned} {r^p} & = \exp \left[- {\sigma _p} {\sum \limits_j}{||{{\hat{p}}_t^j}-p_t^j||}^2\right] \\ {r^e} & =\exp \left[ - {\sigma _e} {\sum \limits _i} {|| {{\hat{x}}_t^i}-{x_t^i}||}^2\right] \end{aligned}$$
  where *j* is the index of joints, (${\hat{p}}_t, {\hat{x}}_t$) are the reference joint and end-effector positions.
- Root Reward ($r_{root}$): This reward aims to track the task root motion including the root’s position ${\hat{x}}_t^{root}$ and rotation ${\hat{q}}_t^{root}$.
  11 $$\begin{aligned} {r_t}^{root} = \exp [-\sigma _{r1}{||{{\hat{x}}_t}^{root}- {x_t}^{root}||}^2-{\sigma _{r2}}{||{{\hat{q}}_t}^{root}-{q_t}^{root}||}^2] \end{aligned}$$
- CoP (Center of Pressure) Reward ($r_{CoP}$) [17]: This reward is to encourage the controller to predict an action that will improve the balance and robustness of the exoskeleton’s motion. The movement of system CoP is an important indicator of system stability and balance, and this reward is to motivate the current CoP position $c_t^{cop}$ to stay inside a stable region *S* around the center of the foot support. By considering the geometry of the foot in the LLRE design (the width and length of the foot are 12 cm and 30 cm), the stable region for foot CoP is defined as a smaller rectangle area *S* around the foot geometric center whose width and length are set to 7 cm and 11 cm respectively (narrower in the lateral direction than forward direction). And the CoP reward function is expressed as
  12 $$\begin{aligned} {{r_t}^{cop}}=\left\{ \begin{aligned} & \exp [- {\sigma _{cop}}{||D({c_t}^{cop}, S)||}^2], \quad \text {if} \, {{c_t}^{cop}} \in S\\&0, \quad \text {if}\, {{c_t}^{cop}}\notin S\\ \end{aligned} \right. \end{aligned}$$
  where $D(\cdot , \cdot )$ is the Euclidean distance between CoP and the center of *S*.
- Action Smoothness Reward ($r_{as}$): This reward encourages smooth action prediction by penalizing the second-order finite difference derivatives of the actions.
  13 $$\begin{aligned} r_{as} = \exp [-\sigma _{as}||(a_s)_t - 2(a_s)_{t-1} + (a_s)_{t-2}||^2] \end{aligned}$$
- Foot clearance Reward ($r_{fc}$): This reward penalizes the roll and pitch angles of the swing foot to encourage the foot to stay parallel with the ground and create more foot clearance to avoid tripping.
  14 $$\begin{aligned} r_{fc} = \exp [-\sigma _{fc}||sin(\theta _{roll,pitch})||^2] \end{aligned}$$
- Torque Reward ($r_{\tau }$): This reward is to reduce energy consumption and to improve efficiency and prevent overburdening joint actuators.
  15 $$\begin{aligned} r_{\tau } = \exp \left[ -\sigma _{\tau }\sum _i||\tau _i||^2\right] \end{aligned}$$
  where *i* is the index of actuated joints.

The output of the neural network predicts the joint target positions. To obtain smooth motions, the output from the control policy network is first processed by a second low-pass filter before being applied to the LLRE. Moreover, we apply preprocessed actions (output) that are linearly interpolated from two consecutive filtered actions during each time step. We consider the actuator torque limit in the simulation, the preprocessed actions $a_{s,t}$ are specified as PD targets and the final PD-based torques applied to each joint are calculated as

16 $$\begin{aligned} \tau = clip\left( k_p(a_{s,t}-p_{t})-k_v\dot{p}_t, - {\hat{\tau }}, {\hat{\tau }} \right) \end{aligned}$$

where $p_t$ denotes the joint angle of the LLRE. $k_p$ and $k_v$ are the proportional gain and differential gain, respectively. The function $clip(\cdot )$ returns the upper bound ${\hat{\tau }}$ or the lower bound $-{\hat{\tau }}$ if the torque $\tau$ exceeds the real actuator torque limit.

### Learning with proximal policy optimization (PPO)

An effective solution to many RL problems is the family of policy gradient algorithms, in which the gradient of the expected return with respect to the policy parameters is computed and used to update the policy parameters $\theta$ through gradient ascent during training. To train the RL networks proposed here, we use the state-of-the-art RL algorithm known as Proximal Policy Optimization (PPO), a model-free policy gradient algorithm that samples data through interaction with the environment and optimizes a “surrogate” objective function [33]. It utilizes a trust region constraint to force the control policy update and ensure that the new policy is not too far away from the old policy. The probability ratio $r_t(\theta )$ is defined by:

17 $$\begin{aligned} r_t(\theta )= \frac{\pi _\theta (a_t|s_t)}{\pi _{\theta _{old}}(a_t|s_t)}. \end{aligned}$$

This probability ratio is a measure of how different the current policy is from the old policy $\pi _{\theta _{old}}$ (the policy before the last update). A large value of this ratio means a large change in the updated policy compared to the old one. PPO also introduces a modified objective function that adopts clipped probability ratio which forms a pessimistic estimate of the policy’s performance and avoids a reduction in performance during the training process. The following “surrogate” objective function by considering the clipped objective is applied to update the policy parameters.

18 $$\begin{aligned} L(\theta )= \mathbb {E}_t \left[ min \left( r_t(\theta ){\hat{A}}_t, clip(r_t(\theta ), 1-\varepsilon , 1+\varepsilon ){\hat{A}}_t \right) \right] \end{aligned}$$

where $\varepsilon$ is a small positive constant which constrains the probability ratio $r_t(\theta )$. ${\hat{A}}_t$ denotes the advantage value at time step *t*. The advantage value ${\hat{A}}_t$ is a measure of how much a certain action is a good or bad decision given a certain state. It is defined as the discounted rewards *R* minus the predicted value *P*. The discounted reward *R* is the weighted sum of all the rewards during each time step of the current episode. The predicted value *P* is the estimated final return in this episode starting from the current state. If ${\hat{A}}_t$ is positive, it means the action taken by the controller is good and a positive reward is obtained by taking the action. So the algorithm improves the probability of this action. On the other hand, if ${\hat{A}}_t$ is negative, then the algorithm needs to decrease the action probability. For more details, please refer to [33]. $clip(\cdot )$ is the clipping function. Clipping the probability ratio discourages the policy from changing too much and taking the minimum results in using the lower, pessimistic bound of the unclipped objective. Thus, any change in the probability ratio is included when it makes the objective worse, and otherwise it is ignored. This can prevent the policy from changing too quickly and leads to more stable learning. The control policy can be updated by maximizing the clipped discounted total reward in Eq. 18 with a gradient ascent.

### Human muscle coordination via RL and supervised learning

Predictive human muscle-actuated simulations based on deep RL have achieved some remarkable results [29, 34]. For example, the simulation presented in [29] consists of a high-fidelity human musculoskeletal model that represents the detailed joints and muscles around the upper and lower extremities and a deep neural network RL-based muscle-actuated controller. It can simulate many aspects of human motions using deep RL, such as steady walking, running, even jumping in a predictive manner. Inspired by their work [29] and to further incorporate realistic human-exoskeleton interactions for the purpose of controlling a LLRE, this paper designs a decoupled network structure that combines the LLRE control policy network with an RL-based interaction network and a supervised learning-based muscle coordination network for muscle-actuated control (A in Fig. 2) in the human-exoskeleton interaction environment.

#### Human-exoskeleton interaction network

The human-exoskeleton interaction network within the RL framework aims to produce a stochastic control policy $\pi _{\phi }(a_h|s_{human})$ that predicts the target human joint poses $a_h$ given human skeletal states $s_{human}$, where $\phi$ denotes network parameters to be optimized using PPO. The network structure is the same as the motion imitation network in the LLRE control loop in Fig 2. We design the reward of this network to encourage the control policy to minimize interaction forces between the human and the LLRE, considering the patient’s desire to follow the exoskeleton movement and reduce strap pressure on the body, as follows.

19 $$\begin{aligned} r_{int} = \exp \left[ -\sigma _{int}\sum _i||f_i||^2\right] \end{aligned}$$

where $f_i$ is the *i*th bushing interaction force between the human and exoskeleton.

#### Muscle coordination network

The interaction network predicts desired human joint angles during human-exoskeleton interaction, which are fed to the PD control to compute the desired human joint accelerations $\varvec{\ddot{q}}_d$. The muscle coordination network is constructed to coordinate the activations of all muscles to produce accelerations as close as possible to the desired human joint accelerations $q_d$. From Eq. 6, we have a linear mapping between $\varvec{\ddot{q}}$ and $\varvec{a}$. We encourage the human joint acceleration to track the desired human joint acceleration $\varvec{\ddot{q}}_d$ from the interaction network. Following [29], we formulate this problem into the supervised learning-based regression framework to learn collaboratively with the interaction network and motion imitation network. Let $a = \pi _{\varphi } (\varvec{q}_d, s_{muscle})$ be a network policy that maps desired human joint torque to muscle activations $\varvec{a}$. The muscle state $s_{muscle} = (vec(\varvec{A}), \varvec{e})$ is defined to encode the information that converts muscle activations into muscle actuated joint accelerations. The loss function to minimize the discrepancy between the desired and actuated joint acceleration is designed as follows:

20 $$\begin{aligned} loss = {\mathbb {E}}||\varvec{\ddot{q}}_d-\varvec{Ka(\varphi )}-\varvec{b}||^2+w_{a}||\varvec{a}||^2 \end{aligned}$$

where $w_a$ is a weight. The muscle coordination network is implemented as a MLP network that consists of four fully connected layers. We use both the *tanh* and ReLU nonlinear function at the output layer to enforce the muscle activations in the normalized range [0, 1]. To solve this regression network to minimize the loss function in Eq. 20, it needs to sample a large collection of tuples $\varvec{(K, b, A, e)}$. Because the motion imitation network generates numerous episodes during training, we can sample the muscle tuples for regression from the episodes.

To elucidate the offline learning process of the proposed method, the pseudocode is provided in Algorithm 1. During learning, this proposed control scheme alternates between the motion imitation network, interaction network, and muscle coordination network to collect tuples and jointly update the policy parameters. One of the unique advantages of this decoupled structure is that it allows each network to operate separately with distinct focuses. For example, the LLRE control policy network focuses on hardware control with only proprioceptive information input. As a result, the trained policy can be readily deployed on the physical exoskeleton without the need of human sensing.
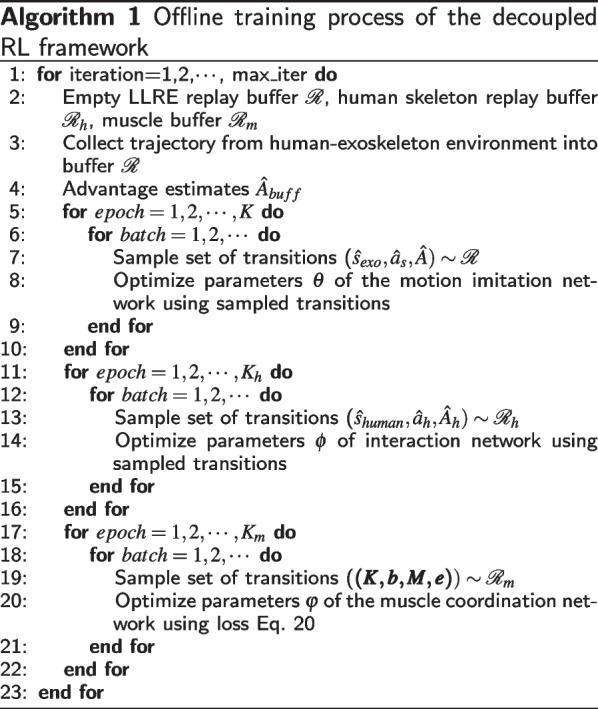


#### Dynamics randomization for the exoskeleton and muscle strength

To reduce the discrepancies between the motor control in the simulation and the real hardware, we utilized the dynamic randomization method to consider the mechatronic characterization of the real actuator in the simulation. The dynamic randomization method is a popular technique in machine learning-based control for sim-to-real transfer (transferring trained control policies from simulation to the real world) [35–37]. Instead of training a policy in a single environment with fixed robot dynamics, dynamic randomization varies the robot dynamics in the high-level or low-level motor control during training, thereby encouraging the control policy to learn robust strategies that are functional across different exoskeleton dynamics. The domain randomization employed in this study enables the controller to handle the sim-2-real differences for the transfer or deployment to the real hardware.

To account for motor control inaccuracy (e.g. the low-level motor control may not be able to faithfully execute the commanded torque from Eq. 16) and control delay on the hardware, we chose to randomize the torque command tracking accuracy and control delay in the dynamic randomization (shown in Table 2). The motor torque command tracking accuracy is randomized with a range [0.8,1,2] as listed in Table 2. Given a computed torque command $\tau$, the torque applied to the joints will be randomly sampled in a range [0.8,1.2]*$\tau$ in the simulation. For the control delay, we choose a randomization range [0,0.04]s, with an upper bound at 0.04 s that simulates possible low bandwidth and slow step response of the motors in the physical exoskeleton system. During our testing, the controller is robust against the command tracking accuracy within the range of [0.7, 1.3] and against control delay within the range of [0,0.06 s] (both listed in Table 2) Moreover, to account for the delay in sensor data transmission and reading, we add the dynamic randomization of the observation latency within a range [0, 0.04]s during the training. This randomization simulates the sensor noise and time delay during information transfer, which can make the trained controller more robust against real noise and delay from the physical exoskeleton. The Dynamixel Pro motor has sufficiently fast mechanical and electrical response times and an onboard encoder that has a latency well below 0.04 s, so the range [0, 0,04]s can sufficiently cover possible variance. Considering the observation latency, motor command tracking accuracy, and control delay improves the reality of the simulations and further increases the difficulty of the control policy training.

Another key challenge in developing a robust walking controller for a LLRE is to deal with patients with different degrees of disability, which are often manifested by muscle weakness or paralysis. To generate a universal walking control policy that has strong robustness against different magnitudes of human interactions forces without the need of tuning of control parameters, we must consider various muscle conditions of the patient and incorporate these conditions into the virtual environment. Here we propose to incorporate a novel muscle strength randomization process into the training workflow. Although muscle weakness or paralysis can be caused by a variety of neuromuscular disorders that affect different physiological properties of the muscle, we choose to randomize the maximum muscle isometric forces $F_{max}$ in Eq. 1 to achieve the end result of limiting the muscle’s capability to generate force. Scaling the maximum isometric forces from 1 to 0 decrease the force generation capacity of the muscle from full capacity to full paralysis. By simply scaling the maximum isometric forces of all muscles or selected muscles (e.g. on one side of the body) within prescribed ranges, we can simulate different conditions of disability such as muscle weakness, hemiparesis, and full paralysis. From our numerical experiments, we find that training with randomized muscle strength is critical to learn robust walking behavior that can handle varying human-exoskeleton interaction forces and consequently produce a LLRE controller that uses only proprioceptive information from the exoskeleton itself.

#### Online deployment process of the controller

The Dynamixel Pro smart actuators used for all exoskeleton joints contain their own microprocessor (ARM CORTEX-M4 (32Bit)) and angular encoders. The motors are connected to a mini-PC (fitPC CompuLab IPC2) through a USB2Dynamixel connection. The motor encoders output the joint angle and angular velocities of the hip, knee and ankle joints, and data are transmitted to the mini-PC with RS485 protocol. The load cell force sensors are linked to a small DAQ card and connected to the mini-PC through a USB cable, with data transmitted via a serial port. Matlab programs and mex functions in C++ are used for the overall control by reading motor encoder and force sensor data and sending out control commands through USB connections.

For the high-level neural network-based controller, after acquiring a parameter set of a trained policy from our RL training, we can use Algorithm 2 to deploy the learned neural network controller to the physical lower limb rehabilitation exoskeleton system. The three-layers neural network (MLP) structure (same as the simulation) is built on Matlab, and the trained parameter set (weight and bias) of this neural network obtained from the simulation will be ported to the neural network in Matlab. The low-level torque control architecture is composed of an inner-loop and an outer-loop control. The inner loop implements motor current control in the local motor controller. The outer loop implements the torque control in Matlab using feedback signals from motors, loadcells, and encoder-based readings.
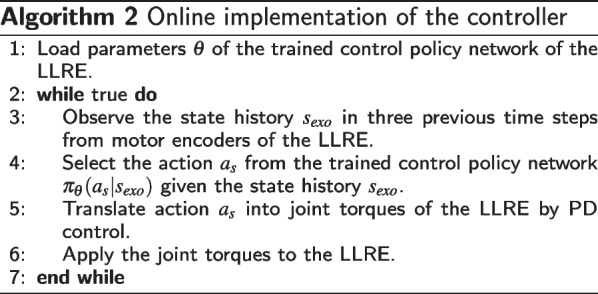


## Numerical experiments

In this section, we first present a learned controller for the LLRE to perform the walking motion without a human and then conduct several numerical experiments involving human subjects with different neuromuscular disorders (healthy, flaccid quadriplegia, muscle weakness, and hemiparesis) to demonstrate the ability of the LLRE to perform robust walking motion under varying human-exoskeleton interactions.

### Model simulation settings

Our control system involves three deep neural networks as described above and the training of these networks relies on the integrated simulation environment that considers the human-exoskeleton interaction, exoskeleton control, and active muscle contraction. During training, the time integration frequency for the environment is 600Hz, and the control frequency (for both exoskeleton and human) is set to 30Hz. The open-source library DART [38] is utilized to simulate the exoskeleton and human skeleton dynamics. The GRFs are computed by a Dantzig LCP (linear complementary problem) solver [39]. The training and testing are performed with a desktop computer with an Intel®Xeon(R) CPU E5-1660 v3 @ 3.00GHz $\times$ 16.

### RL-based controller settings

In this paper, the reference walking motion was manually created based on a human walking motion. The reference motion can provide guidance for motion imitation for the LLRE but needs not to be generated precisely. PyTorch [40] is used to implement the neural networks and the PPO method for the learning process. The networks are initialized by the Xavier uniform method [41]. In total about 20 million samples are collected during training. The policy and value networks of the motion imitation network and interaction network are updated at a learning rate of $10^{-4}$, which is linearly decreased to 0 when 20 million samples are collected. The max iteration is set to 120,000. The learning rate of the muscle coordination network is also set to $10^{-4}$. Hyperparameters settings for training using PPO are shown in Table 1. To verify the robustness of the trained controller, we test the control policies in out-of-distribution simulated environments, where the dynamic parameters of the exoskeleton are sampled randomly from a larger range of values than those during training. Table 2 shows the dynamics randomization parameters of the LLRE and their ranges during training and testing. According to the PD control Eq. 16, the proportional gain $k_p$ and differential gain $k_v$ are set to 900 and 40, respectively. We conducted a grid search of weights in a proper range. The weights of the rewards can be considered as the hyperparameters of neural networks. Grid search is a tuning method that attempts to compute the optimum values of hyperparameters. It is an exhaustive search that is performed on a specific setting of hyperparameters of a model. For example, $w^p$ is chosen from [0.25, 0.5, 0.75, 1], $w^{ee}$ and $w^{root}$ are chosen from [0.1, 0.2, 0.3,…,0.9,1]. We measured the overall reward according to Eqs. 10- 15 with different sets of weights and chose the following set of weights with the best overall performance during the testing: $w^p= 0.75, w^{ee} = 0.4, w^{root} = 0.4, w^{as} = 0.3, w^{fc} = 0.2, w^{\tau } =0.1$, $w_a = 0.1$, $w^{cop} = 0.06$, $w^{int} = 0.05$. We carried out five numerical experiments to demonstrate that the decoupled RL-based framework is able to generate a universal controller for the LLRE to robustly perform natural walking motion and assist human with various neuromuscular disorders without the need for tuning control parameters.

**Table 1:** Hyper-parameters settings for training

Parameters	Value	Parameters	Value
Discount factor	0.99	Epochs	10
Policy Adam learning rate	$10^{-4}$	Clip threshold	0.2
Batch size	128	Memory buffer	2048

**Table 2:** Dynamics randomization details of LLRE during training and testing

Dynamic parameters	Training range	Testing range
Friction coefficient	[0.9,1.6]*default value	[0.7,2.0]*default value
Mass	[0.8,1.2]*default value	[0.7,1.5]*default value
Motor command tracking accuracy	[0.8,1.2]*default value	[0.7,1.3]*default value
Observation latency	[0,0.04]s	[0,0.06]s
Control delay	[0,0.04]s	[0,0.06]s
Inertial	[0.5,1.5]*default value	[0.4,1.6]*default value
Center of mass	[0.9,1.2]*default value	[0.8,1.3]*default value

**Fig. 4:**
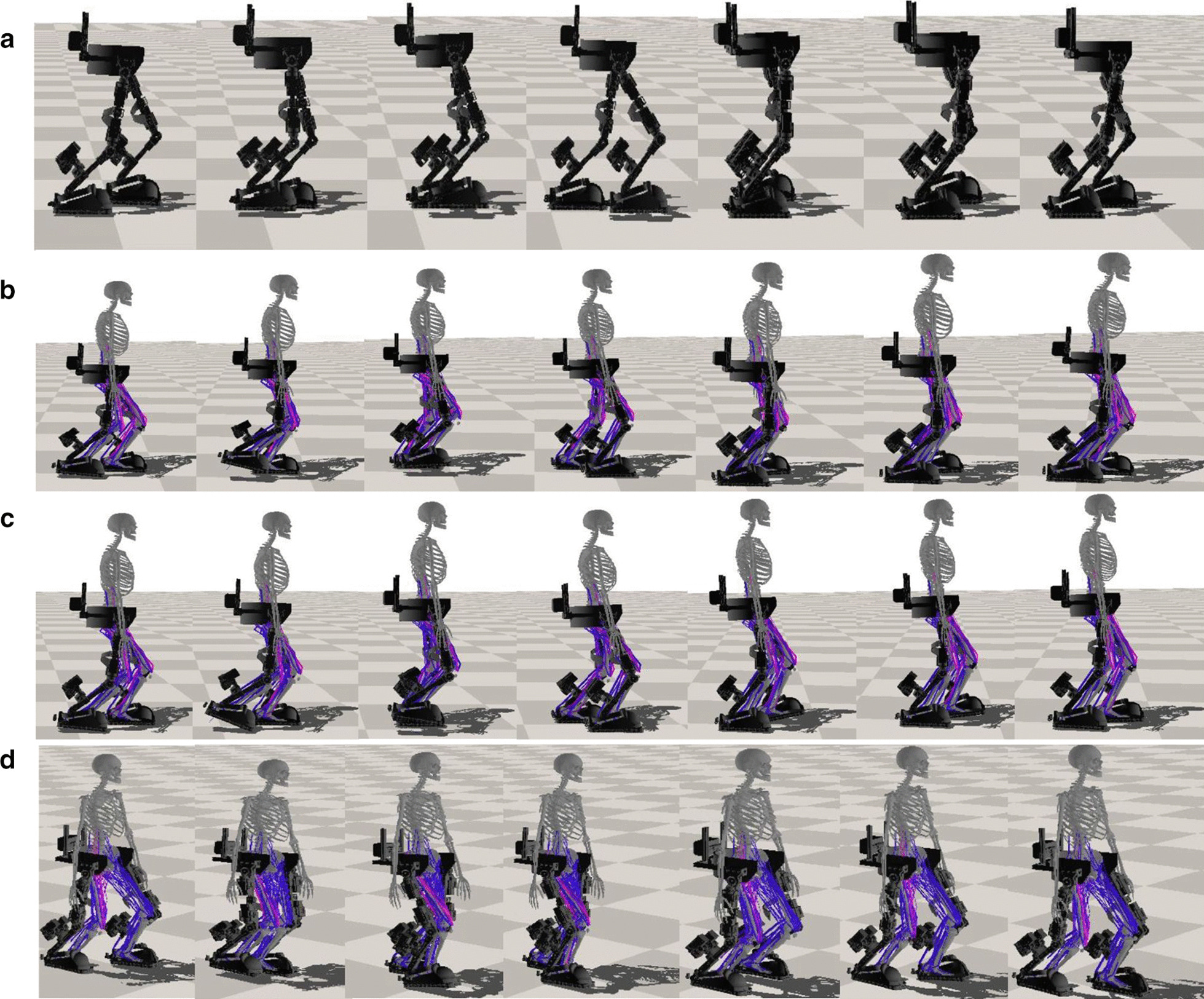
Snapshots of the walking control of the LLRE. The learned controller trained from the decoupled RL-based control framework enables the LLRE to perform walking assistance under varying human-exoskeleton interactions from human subjects with various neuromuscular disorders. **a** Autonomous walking control without human involved. **b** robust walking control with a fully healthy, muscle-actuated human. **c** robust walking assistance with a human with muscle weakness. **d** robust walking assistance with a human with left hemiparesis. The color of the muscle indicates its activation, with purple being the highest and blue being the lowest

### Numerical experiments and results

#### Walking without human-exoskeleton interactions

**Fig. 5:**
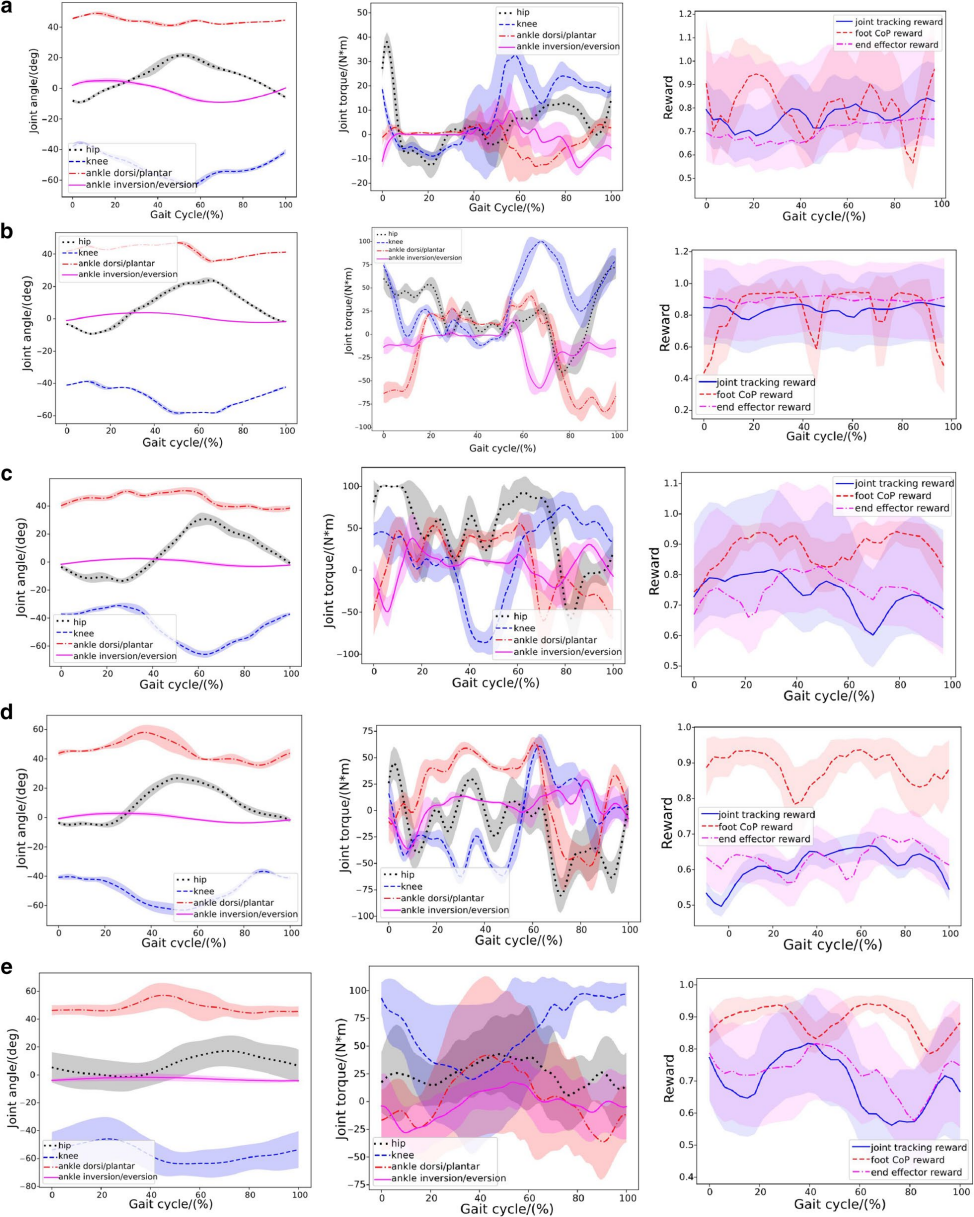
Statistical analysis results during 40 walking cycles without human or with human subjects under different neuromuscular conditions (curve: mean; shade: STD). From left to right are exoskeleton joint angles, joint torques, and rewards, including the joint position tracking and end-effector reward in Eq. 10 and the CoP reward in Eq. 12. **a** without a human; **b** with a passive human; **c** with a healthy human; **d** with a human with muscle weakness; **e** with a human under the left hemiparesis condition

In the first case, we first validate the robust walking motion learned from the controller of the LLRE without human-exoskeleton interactions. We only train the motion imitation network (the LLRE control loop in Fig. 2) for the LLRE to imitate the reference walking motion without the human involved. A series of snapshots of the walking motion resulting from the learned control policies can be observed in Fig. 4a. The learned controller of the LLRE is able to perform balanced walking motion autonomously. Figure 5a represents the joint behavior statistics of the hip flexion/extension, knee flexion/extension and ankle dorsiflexion/plantarflexion joint angles during 40 walking cycles. The corresponding joint torques are depicted in Fig. 5a. The joint angles and torques are relatively smooth. To further validate the learned controller’s ability to cope with uncertain dynamics of the LLRE, we test the learned controller in 200 out-of-distribution simulated environments, where the dynamics parameters are sampled from a larger range of values than those used during training (shown in Table 2). The third figure in Fig. 5a visualizes the performance of the learned controller in 200 simulated environments with randomized dynamics. It depicts the reward statistics (mean and standard deviation) with respect to time under 200 simulated environments with different dynamics. The joint position tracking (Eq. 10) and foot CoP (Eq. 12) also achieve a high reward under more diverse dynamics of the LLRE. The end-effector reward indicating the foot tracking performance maintains a high value, revealing the LLRE can perform a stable walking motion without falling in 200 simulated environments with unfamiliar dynamics. These results demonstrate that the learned controller is able to effortlessly generalize to environments that differ from those encountered during training and achieve good control performance under very diverse dynamics.

#### Walking with a passive/quadriplegic human

In this case, we investigate the performance of the learned controller under the human-exoskeleton interactions from a passive human (e.g. a quadriplegic patient). Linear bushing forces are utilized to simulate the interaction between the human and LLRE. In this particular case, we do not consider the active muscle contraction of the human operator or actuation torques produced from human joints, assuming the operator could be a patient suffering from SCI or severe stroke, with very limited or no control of his or her own body. Thus, only the passive muscle forces in Eq. 1 during movement are incorporated. The walking assistance learned by the LLRE and the performance of the motion controller are shown in Fig. 5b.

Figure 5b displays the statistical results of the hip, knee, ankle joint angles and torques generated by the learned controller during 40 walking cycles. We can clearly observe that the torques calculated from the PD control are still smooth under human-exoskeleton interactions.

Reward statistics of the controlled LLRE in 200 simulated environments are shown in the third figure of Fig. 5b. The high joint tracking reward and end-effector tracking reward indicate that the learned controller has strong stability and robustness to the varying human interaction forces from a passive human. This case demonstrates the capability of the LLRE to carry a passive human to perform the walking assistance with robustness.

#### Walking with a healthy human

**Fig. 6:**
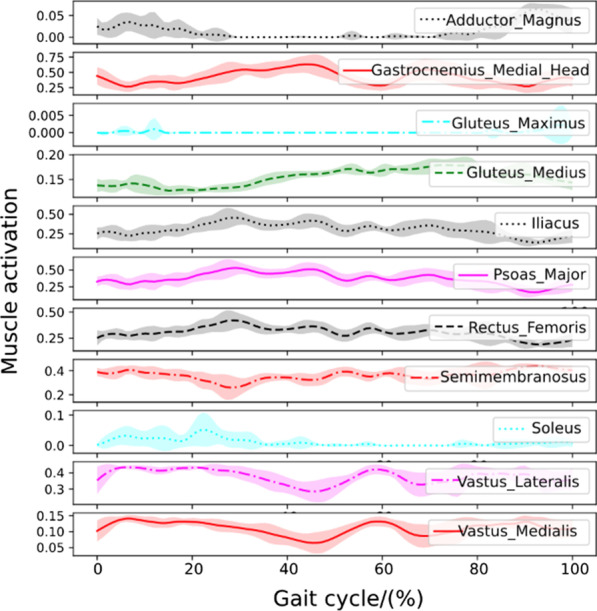
Major muscle activations on the right leg when performing the skill with a fully healthy human

To test the controller’s robustness under active muscle-actuated human interaction forces, we design a numerical experiment where the human is fully muscle-actuated and has no disability. For simplicity, we only activate the 162 lower leg musculotendon units while ignoring the active contraction of the upper body muscles. A series of snapshots of the walking assistance resulting from the learned control policy can be observed in Fig. 4b. Statistical results of joint and torque trajectories with human-exoskeleton interaction from an active human are shown in Fig. 5c. Reward statistics of the controlled LLRE during 40 walking cycles are shown in the third figure in Fig. 5c. The statistical results of the muscle activations of major lower-limb muscles on the right side body are illustrated in Fig. 6. Muscle activations predicted from the muscle coordination network show smooth patterns. The hamstring and gastrocnemius muscles are the main muscles responsible for knee flexion and ankle dorsiflexion and plantarflexion. The activation pattern of the gastrocnemius is also consistent with the result using electromyogram measurements [42], in which the gastrocnemius developed a high activation during the stance phase.

**Fig. 7:**
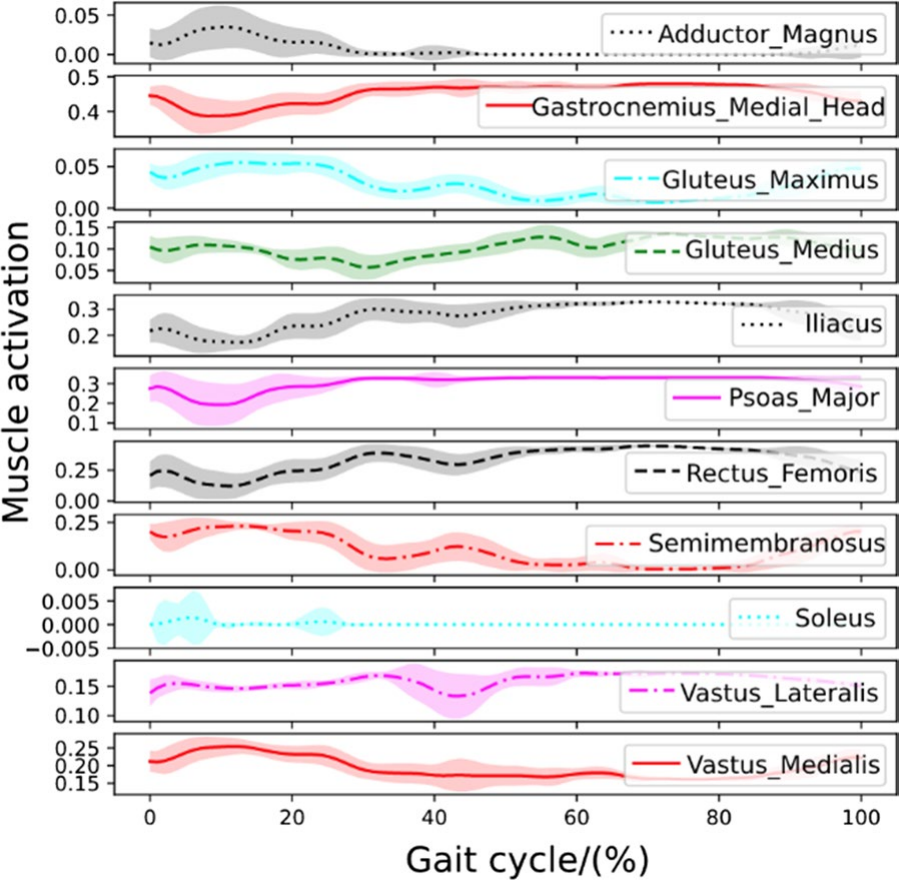
Major muscle activations on the right leg when performing the skill with a patient with muscle weakness

**Fig. 8:**
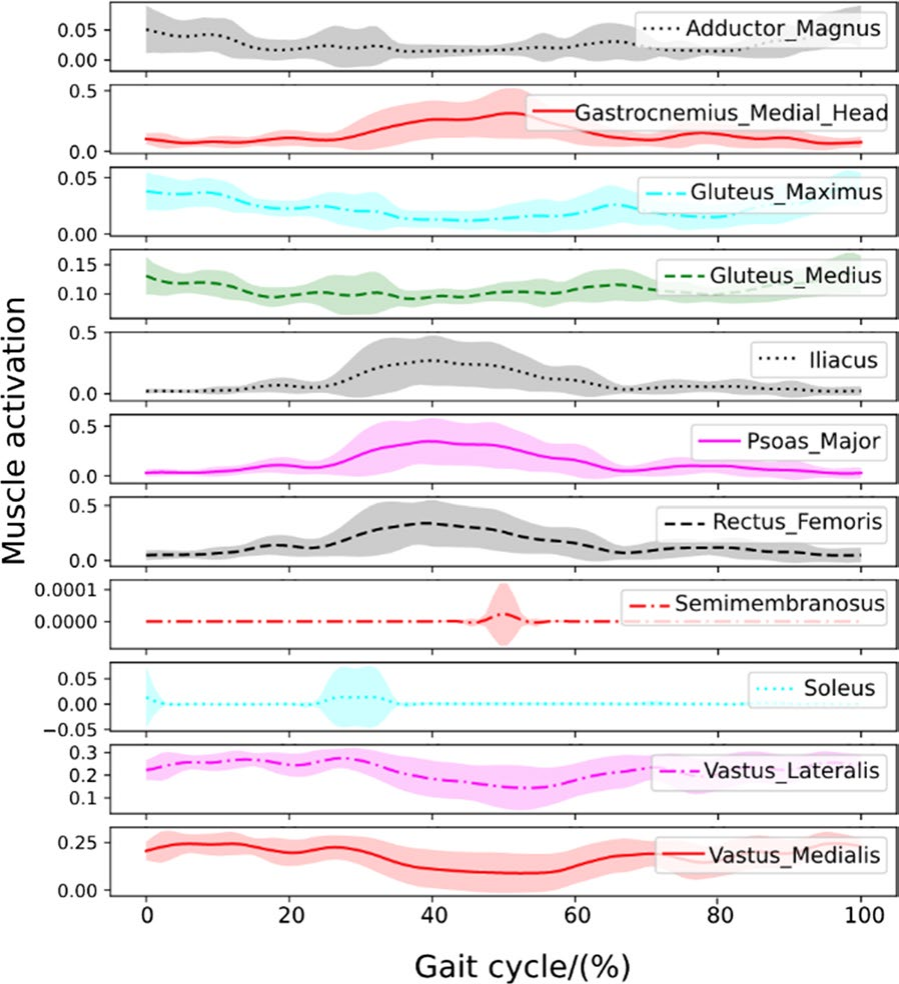
Major muscle activations on the right leg when performing the skill with a hemiplegic patient

#### Walking with a human with muscle weakness

Muscle weakness has been considered to be a minor modifiable risk factor for health outcomes, and it plays a significant role in the etiology of disability [43]. It could be caused by age-related loss of muscle mass such as dynapenia or loss of muscle strength due to neuromuscular disorders. In this case, muscle weakness is incorporated into our human model by reducing all muscles’ force generation capability by half. A series of snapshots of the walking assistance resulting from the learned control policy can be observed in Fig. 4c. Figure 5d shows the Joint behavior statistics with muscle weakness during multiple cycles. Statistical results of the muscle activations of major lower-limb muscles on the right side of the body are shown in Fig. 7. Muscle activations predicted for this case have bigger variances than those in the fully healthy human case. This case successfully validates that the learned controller can generate robust walking motion assisting a patient with muscle weakness condition.

#### Walking with a hemiplegic patient

Hemiparesis due to stroke impairs a patient’s ability to walk. The disabilities caused by the hemiparesis, together with the ensuing safety concern, prevent many patients from practicing walking by themselves and may contribute to a further decline in their walking ability or physical condition. It has been reported that following a stroke, patients often suffer from impaired balance control [44]. In this case, we will demonstrate that the learned controller is capable of providing the assistance to help a hemiplegic patient (on the left side) perform robust walking assistance. A series of snapshots of the walking assistance resulting from the learned control policy can be observed in Fig. 4d. Statistical results of joint angles and torques with the hemiplegic patient are shown in Fig. 5e. Statistical results of the muscle activations of major lower-limb muscles on the right side are illustrated in Fig. 8. The muscle activation results show even greater variances than those in the previous two human cases. Simulation videos of the LLRE assisting users with different disabilities such as passive muscles (quadriplegic), muscle weakness, and hemiplegic conditions are included in Additional file 1.

### Robustness and gait symmetry

We aim to control the exoskeleton to walk similar to a provided normative trajectory (i.e. track a reference joint motion) with strong robustness and sound gait symmetry. To quantify the controller’s performance on tracking and walking stability, we examine several different aspects of the results, including tracking accuracies of joint trajectories and foot CoP. In the third column of Fig. 5a–e, we present the reward statistics of the controller including the joint motion tracking reward and CoP balance reward under 200 simulated environments with different dynamics. The high joint tracking reward and CoP balance reward verify the effectiveness and robustness of the resultant walking controller in assisting users with different disabilities.

**Fig. 9:**
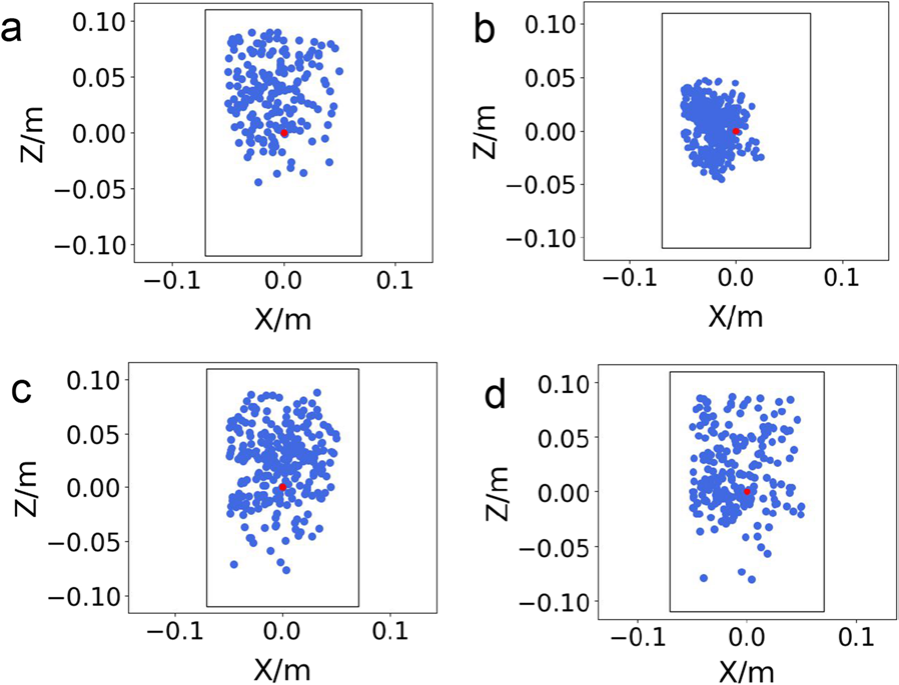
CoP-based stability analysis. It shows the CoP locations under different human conditions during 100 walking gait cycles. **a** Fully passive; **b** healthy; **c** muscle weakness; and **d** hemiparesis. We can clearly see that all CoP positions stay inside in the stable region during the walking motion

Moreover, we present the root mean square error (RMSE) of joint tracking accuracy and CoP-based stability analysis to further demonstrate the strong performance of the controller as shown in Table 3 and Fig. 9. From Table 3, the relatively low RMSE of the hip, knee, and ankle angle tracking in all cases demonstrates good joint tracking accuracy of the controller. Figure 9 shows the CoP-based stability analysis. It shows the CoP locations under different human conditions during 100 walking gait cycles. We can clearly see that all CoP positions stay inside in the stable region during the walking motion. Note in Fig. 5, we observe that the predicted joint torques are mostly well below 100 Nm, smaller than the continuous actuation torque (132 Nm) operating at around 9.3 A (continuous current). This shows we can achieve the desired robustness with relatively low current consumption. However, if we increase the weight for the imitation reward or decrease the weight for the torque reward, it will likely result in a controller with better tracking accuracy but higher current consumption.

**Table 3:** Root mean square error (RMSE) for joint tracking accuracy

Case	Hip (deg)	Knee (deg)	Ankle Dorsi/plantar (deg)	Ankle Inver/ever (deg)
Passive	2.048	3.437	4.297	2.406
Healthy	3.076	5.294	4.583	2.807
Muscle weakness	4.870	5.157	4.437	3.258
Left hemiparesis	4.492	4.309	5.082	4.462

To quantify human gait symmetry when walking with the exoskeleton, we use the ratio index $R=X_R/X_L$ [45] to qualify the gait symmetry between the right and left legs. $X_R$ and $X_L$ denote the mean joint angle of the right leg and the left leg, respectively. If the value of the ratio index is close to 1, the human shows a good symmetry gait pattern. Table 4 shows the gait symmetry analysis of the hip, knee, ankle joints for the four simulation cases involving human subjects with different neuromuscular conditions (fully passive, healthy, or quadriplegic, muscle weakness, and hemiparesis). As it can be observed from the table, the case for the human with left hemiparesis condition has the highest asymmetry index for most joints except the knee, whereas the healthy subject exhibits better symmetry for most joints except the knee. For the left hemiparesis case, the hip, knee, and ankle angles of the right leg are all greater than those of the left (paraplegic) leg. The walking assistance from the LLRE certainly improves the gait symmetry of the hemiplegia human compared to that without exoskeleton, but the asymmetry is still evident due to vastly different neuromuscular conditions on the two sides. Nonetheless, if the neuromuscular condition improves with more rehabilitation training, we expect the gait symmetry to continuously improve as well.

**Table 4:** Gait symmetry analysis

Case	Hip	Knee	Ankle dorsi/plantar	Ankle inver/ever
Passive	0.86	1.08	0.95	0.78
Healthy	0.92	1.12	1.04	0.86
Muscle weakness	0.82	1.06	0.94	0.56
Left hemiparesis	1.49	1.03	1.08	1.46

**Fig. 10:**
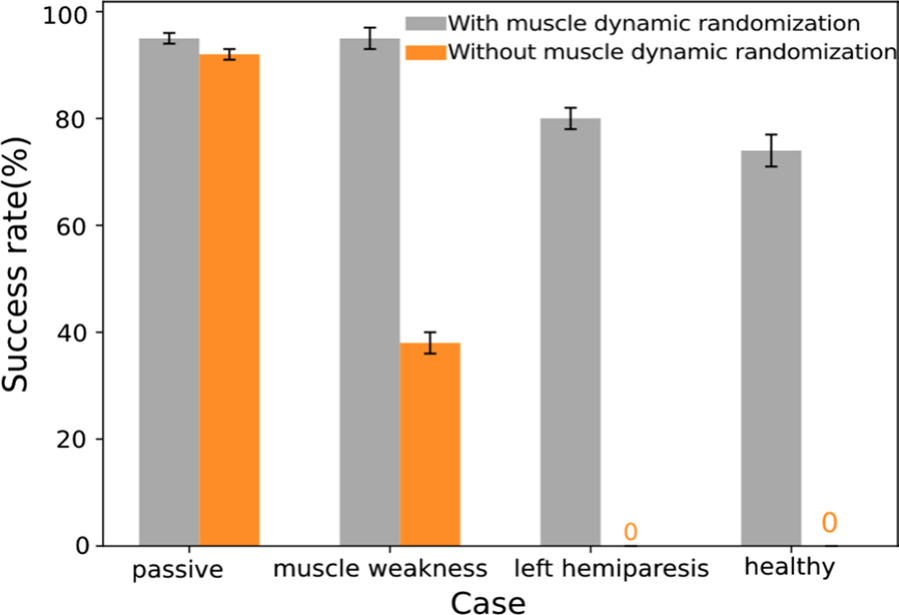
Ablation study. It shows the comparison results of success rates for different human conditions with/without muscle strength randomization training. The success rate is evaluated over 100 trials for each condition. The success rates in all human conditions are significantly higher than that trained without muscle strength randomization

**Fig. 11:**
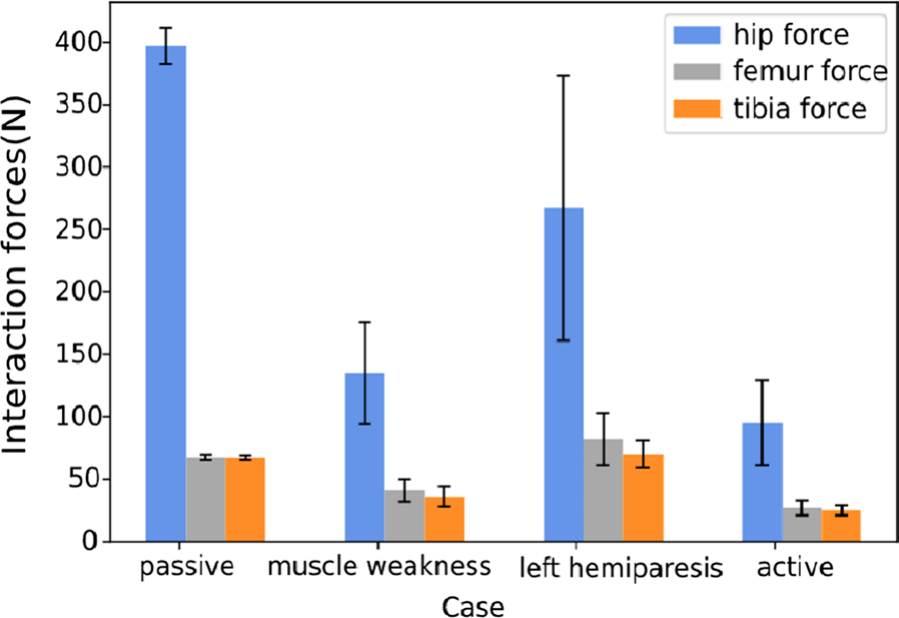
Human-exoskeleton interaction (strap) forces under different cases during 40 walking cycles

Since a good indication of balance and robustness for walking is to avoid falling, we conducted an ablation study to show the success rate (SR) of control policies trained differently. Figure 10 summarizes the SR of trained policies with/without muscle strength randomization, for which each case is evaluated over 100 walking trials (40 cycles each). If we train the neural network under the passive human case, the trained control policy has poor robustness to handle the other three conditions, with less than 40% SR for the muscle weakness case and zero SR for both the left hemiplegia and healthy cases. In contrast, if the policy is trained with muscle strength randomization, it has high SR for all cases. The success rates under the human with left hemiplegia condition are significantly lower when trained without the muscle strength randomization, as shown in Fig. 10. Note that the SR is evaluated with test cases that are randomized from larger ranges for physical parameters of the LLRE (Table 2) and therefore some failures are expected.

To study the range of human-exoskeleton interaction forces, we provide in Fig. 11 the strap forces under different cases during 40 walking cycles. The human-exoskeleton strap forces on hip, femur and tibia locations from a passive human are greater than those from muscle weakness, left hemiparesis and healthy human. The hip forces for the passive and left hemiparesis human are significantly higher than the other two cases, which is understandable since either one or two legs can not provide any support to the body weight. Among the four cases, the strap forces are the smallest for the healthy case and the values are all below 100 N. Since the interaction network aims to minimize the interaction forces (Eq. 19), it encourages the healthy human to follow the motion of the exoskeleton to the best of capability and thus produces a low level of interaction forces.

## Discussion

Designing a robust walking controller for a LLRE is particularly important for rehabilitation and represents a crucial challenge due to the safety concerns for the patients. The risk of testing on real humans is even greater, and the cost of testing is often high. The decoupled RL-based neural network architecture proposed in this work incorporates the muscle-actuated human control into the training process and considers realistic effects of a LLRE on the human’s musculoskeletal system in the simulation environment, resulting in an extendable framework to investigate the control of LLREs with the varying human-exoskeleton interactions. From our numerical experiments, the learned controller of the LLRE is capable of providing robust walking assistance to humans with a variety of neuromuscular conditions, such as healthy, passive, or quadriplegic, muscle weakness, and hemiplegic conditions.

The decoupled control structure integrates three deep neural networks, including the motion imitation network for the LLRE, the human-exoskeleton interaction network, and the muscle coordination network for the human muscle control. These three networks are jointly trained in the simulation while they interact with each other to achieve maximum rewards during reinforcement learning. There are several distinct advantages with this decoupled learning structure. First, three separate networks allow independent control of the LLRE, human joint, and muscles. As demonstrated by the cases with and without a human, the same framework can be flexibly utilized for training in all cases by enabling or disabling individual networks. This decoupled network structure also has a unique advantage for sim-to-real transfer, for which only the trained controller of the LLRE need to be deployed on the physical rehabilitation exoskeleton. Second, the control structure can easily incorporate a human or patient with different disability conditions, as demonstrated by all the human cases above.

In this work, only muscle weakness and paralysis resulting from a variety of neuromuscular disorders have been considered to test the robustness of the LLRE controller. Nonetheless, we believe we are able to extend the current work for further investigations on patients with other pathologic conditions such as muscle contracture, muscle spasticity, cerebral palsy, and femoral anteversion. Such an extension will require further adjustment or randomization of muscle parameters such as optimal fiber lengths for contracture [46] or muscle contraction intensity for spasticity [47].

The reference walking motion used in this study was manually created based on a human walking motion, but with the knee angle bent more than the natural human motion. By doing so, we are able to make the walking motion more stable (e.g. with a disabled human or without a human subject), similar to motions generated for some bipedal robots to increase stability. In this case, the reference angle of the knee joint is close to negative 40^o^–60^o^ (flexion, with 0° at straight knee). The controller is able to handle all challenging conditions and walk at a speed close to 1 m/s for all cases. For comparison, the much heavier, self-balancing Atalante exoskeleton can only walk with a subject at a very low speed of around 0.4 m/s [48]. In our current setting, different speeds can be obtained by crafting another reference motion or simply scaling the current motion as the new reference without modifying the controller structure or designing the speed-specific reward functions. Note the reference motion does not need to be perfect or even physically feasible, the RL algorithm with a dynamic simulation environment can auto-correct or modify the motion to be physically feasible while tracking the motion and maximizing all reward functions. The tracking accuracy is able to vary depending on the relative weight of the tracking reward to other rewards.

By incorporating motion imitation into the learning process, the proposed control framework has the capability to potentially learn a diverse array of human behaviors without the need to design skill-specific reward functions. Common rehabilitation motions such as sit-to-stand, walking on inclined ground can be learned by feeding proper target motions. For example, in [17], the authors presented a motion imitation, RL based control of a LLRE for squatting assistance. In this work, we expand its learning framework to include both active muscle contraction and human-exoskeleton interaction force optimization. The current framework is much more general and can handle a variety of motions and humans with different health conditions. Due to the nature of imitation learning, we foresee minimal changes to the current learning framework for different activities, except for crafting different target motions for imitation. The learning process will automatically create specific controllers that can produce physically feasible and stable target motions.

In our current hardware implementation, the control frequency of the lower limb rehabilitation exoskeleton can be set to 100 Hz or lower. We tested the trained control policy in the simulation with different control frequencies between 30 to 100 Hz and were able to obtain robust controllers for all. In this work, we present the control frequency at 30 Hz in the simulation to show the control can be applied at a relatively low frequency, which indicates that the controller is very robust and relies less on the update or control frequency of the hardware (i.e. capable of being applied to motors with low bandwidth or slow step response). From our tests, the use of different control frequencies (30–100 Hz) does not require a different history length, although it is possible to use a different history length for a different frequency (e.g. using the same time window length will cover more history samples at a higher frequency). We found that three time steps of history information (joint positions and velocities of the exoskeleton) are enough to train a desirable controller. A larger number of steps might result in similar or slightly better control performance but will certainly result in a larger neural network model and unnecessary computational load, which is a burden when deployed to the hardware with limited computational power. Therefore, we set the number of time steps to 3 mainly for efficiency. Another benefit of using the same number of history steps is that the trained networks at different control frequencies have the same input and output dimensions and can be used interchangeably for testing in simulations or deployed to the hardware with minimal to no changes.

## Conclusion

This paper proposes a decoupled RL-based control framework for robust walking control of a LLRE system. The framework is flexible enough to train the walking controller with or without human-in-the-loop. It separates the control of the exoskeleton and human voluntary muscle control while integrating the human-exoskeleton interaction in the physics-based simulation environment and trains multiple control networks simultaneously. More importantly, to avoid tuning control parameters to various magnitudes of human-exoskeleton interaction forces or create different LLRE controllers for patients with different conditions of disability, muscle strength randomization is critical in the training process to handle these conditions. Experimental results show that this proposed framework is able to generate a universal, robust walking controller for the LLRE to handle various levels of human-exoskeleton interactions without the need of tuning parameters. The walking controller is shown to be able to provide assistance to healthy humans or patients with different disability conditions including flaccid quadriplegia, muscle weakness, and hemiplegic conditions. The decoupled network structure also allows us to isolate the LLRE control policy network for testing and facilitate straightforward sim-2-real transfer since it uses only proprioception information of the LLRE as the input. In the future, we plan to deploy the trained walking control policy to the physical LLRE with sim-to-real transfer and validate its performance on the real physical system with patients involved.

## Supplementary Information

**Additional file 1.** Slides for additional pictures and videos of the lower limb rehabilitation exoskeleton assisting users with different disabilities such as passive muscles (quadriplegic), muscle weakness and hemiplegic conditions.

## Data Availability

The datasets and code generated during and/or analyzed during the current study are not publicly available due to the conditions of the funding source but are available from the corresponding author on reasonable request.

## Abbreviations

LLRE: Lower limb rehabilitation exoskeleton
RL: Reinforcement learning

## References

[1] Pons JL (2010). Rehabilitation exoskeletal robotics. IEEE Eng Med Biol Magaz.

[2] Baud R, Manzoori AR, Ijspeert A, Bouri M (2021). Review of control strategies for lower-limb exoskeletons to assist gait. J NeuroEng Rehab.

[3] Huo W, Mohammed S, Moreno JC, Amirat Y (2016). Lower limb wearable robots for assistance and rehabilitation: a state of the art. IEEE Syst J.

[4] Banala SK, Kim SH, Agrawal SK, Scholz JP (2008). Robot assisted gait training with active leg exoskeleton (alex). IEEE Trans Neural Syst Rehab Engi.

[5] Mergner T, Lippi V (2018). Posture control-human-inspired approaches for humanoid robot benchmarking: Conceptualizing tests, protocols and analyses. Front Neurorobotics.

[6] Vouga T, Baud R, Fasola J, Bouri M, Bleuler H Twiice–a lightweight lower-limb exoskeleton for complete paraplegics. In: 2017 International Conference on Rehabilitation Robotics (ICORR), 2017. p. 1639–45. 10.1109/ICORR.2017.8009483 28814055

[7] Zhang T, Tran M, Huang H (2018). Design and experimental verification of hip exoskeleton with balance capacities for walking assistance. IEEE/ASME Trans Mechat.

[8] Sun W, Lin J-W, Su S-F, Wang N, Er MJ (2020). Reduced adaptive fuzzy decoupling control for lower limb exoskeleton. IEEE Trans Cybern.

[9] Deng M-y, Ma Z-y, Wang Y-n, Wang H-s, Zhao Y-b, Wei Q-x, Yang W, Yang C-j (2019). Fall preventive gait trajectory planning of a lower limb rehabilitation exoskeleton based on capture point theory. Front Inform Technol Electr Eng.

[10] Moreno JC, Figueiredo J, Pons JL, Colombo R, Sanguineti V (2018). Chapter7exoskeletons for lower-limb rehabilitation. Rehabilitation Robotics.

[11] Schrade SO, Dätwyler K, Stücheli M, Studer K, Türk D-A, Meboldt M, Gassert R, Lambercy O (2018). Development of varileg, an exoskeleton with variable stiffness actuation: first results and user evaluation from the cybathlon 2016. J Neuroeng Rehab.

[12] Huang R, Peng Z, Cheng H, Hu J, Qiu J, Zou C, Chen Q Learning-based walking assistance control strategy for a lower limb exoskeleton with hemiplegia patients. In: 2018 IEEE/RSJ International Conference on Intelligent Robots and Systems (IROS), pp. 2018:2280–2285. 10.1109/IROS.2018.8594464

[13] Baud R, Fasola J, Vouga T, Ijspeert A, Bouri M Bio-inspired standing balance controller for a full-mobilization exoskeleton. In: 2019 IEEE 16th International Conference on Rehabilitation Robotics (ICORR), pp. 2019:849–854. IEEE 10.1109/ICORR.2019.8779440 31374736

[14] Bionics R Rex Technology. https://www.rexbionics.com/us/product-information/ Accessed April, 2020

[15] Wandercraft: Atalante. https://www.wandercraft.eu/en/exo/ Accessed April, 2020

[16] Androwis GJ, Karunakaran K, Nunez E, Michael P, Yue G, Foulds RA Research and development of new generation robotic exoskeleton for over ground walking in individuals with mobility disorders (novel design and control). In: 2017 International Symposium on Wearable Robotics and Rehabilitation (WeRob), pp. 2017:1–2. IEEE

[17] Luo S, Androwis G, Adamovich S, Su H, Nunez E, Zhou X. Reinforcement learning and control of a lower extremity exoskeleton for squat assistance. Frontiers in Robotics and AI. 2021;8. 10.3389/frobt.2021.702845 PMC8326457 34350214

[18] Chen B, Ma H, Qin L-Y, Gao F, Chan K-M, Law S-W, Qin L, Liao W-H (2016). Recent developments and challenges of lower extremity exoskeletons. J Orthopaedic Transl.

[19] Kumar VC, Ha S, Sawicki G, Liu CK Learning a control policy for fall prevention on an assistive walking device. In: 2020 IEEE International Conference on Robotics and Automation (ICRA), pp. 2020:4833–4840. IEEE

[20] Xiong M Research on the control system of the lower limb rehabilitation robot under the single degree of freedom. In: Applied Mechanics and Materials, vol. 643, 2014, p. 15–20.

[21] Shi D, Zhang W, Zhang W, Ding X (2019). A review on lower limb rehabilitation exoskeleton robots. Chin J Mech Eng.

[22] Hu J, Hou Z, Zhang F, Chen Y, Li P Training strategies for a lower limb rehabilitation robot based on impedance control. In: 2012 IEEE Annual International Conference of the IEEE Engineering in Medicine and Biology Society, 2012. p. 6032–5. 10.1109/EMBC.2012.6347369 23367304

[23] Karunakaran K, Abbruzzese K, Androwis G, Foulds R (2020). A novel user control for lower extremity rehabilitation exoskeletons. Front Robotics AI.

[24] Bayon C, Emmens A, Afschrift M, Van Wouwe T, Keemink A, Van Der Kooij H, Van Asseldonk E (2020). Can momentum-based control predict human balance recovery strategies?. IEEE Trans Neural Syst Rehab Eng.

[25] Gao X, Si J, Wen Y, Li M, Huang H. Reinforcement learning control of robotic knee with human-in-the-loop by flexible policy iteration. IEEE Transactions on Neural Networks and Learning Systems. 2021. 10.1109/TNNLS.2021.3071727 33956634

[26] Bingjing G, Jianhai H, Xiangpan L, Lin Y (2019). Human-robot interactive control based on reinforcement learning for gait rehabilitation training robot. Int J Advan Robotic Syst.

[27] Peng Z, Luo R, Huang R, Hu J, Shi K, Cheng H, Ghosh BK Data-driven reinforcement learning for walking assistance control of a lower limb exoskeleton with hemiplegic patients. In: 2020 IEEE International Conference on Robotics and Automation (ICRA), 2020. p. 9065–71. 10.1109/ICRA40945.2020.9197229

[28] Nunez EH, Michael PA, Foulds R 2-dof ankle-foot system: Implementation of balance for lower extremity exoskeletons. In: 2017 International Symposium on Wearable Robotics and Rehabilitation (WeRob), 2017. p. 1–2. IEEE.

[29] Lee S, Park M, Lee K, Lee J (2019). Scalable muscle-actuated human simulation and control. ACM Trans Graphics (TOG).

[30] Zajac FE (1989). Muscle and tendon: properties, models, scaling, and application to biomechanics and motor control. Crit Rev Biomed Eng.

[31] Thelen DG (2003). Adjustment of muscle mechanics model parameters to simulate dynamic contractions in older adults. J Biomech Eng.

[32] Zhou X, Zheng L. Model-based comparison of passive and active assistance designs in an occupational upper limb exoskeleton for overhead lifting. IISE Transactions on Occupational Ergonomics and Human Factors. 2021;1–19. 10.1080/24725838.2021.1954565, https://www.tandfonline.com/doi/pdf/10.1080/24725838.2021.1954565 PMC8789934 34254566

[33] Schulman J, Wolski F, Dhariwal P, Radford A, Klimov O Proximal policy optimization algorithms. arXiv preprint arXiv:1707.06347. 2017.

[34] Song S, Kidziński Ł, Peng XB, Ong C, Hicks JL, Levine S, Atkeson C, Delp S (2021). Deep reinforcement learning for modeling human locomotion control in neuromechanical simulation. J Neuro Eng Rehab.

[35] Tobin J, Fong R, Ray A, Schneider J, Zaremba W, Abbeel P Domain randomization for transferring deep neural networks from simulation to the real world. In: 2017 IEEE/RSJ International Conference on Intelligent Robots and Systems (IROS), pp. 2017:23–30. IEEE

[36] Peng XB, Coumans E, Zhang T, Lee T-W, Tan J, Levine S Learning agile robotic locomotion skills by imitating animals. 2020. arXiv preprint arXiv:2004.00784

[37] Hwangbo J, Lee J, Dosovitskiy A, Bellicoso D, Tsounis V, Koltun V, Hutter M (2019). Learning agile and dynamic motor skills for legged robots. Science Robotics..

[38] Lee J, Grey M, Ha S, Kunz T, Jain S, Ye Y, Srinivasa S, Stilman M (2018). Liu CK Dart: Dynamic animation and robotics toolkit. J Open Source Software.

[39] Baraff D. Fast contact force computation for nonpenetrating rigid bodies. In: Proceedings of the 21st Annual Conference on Computer Graphics and Interactive Techniques, 1994; p. 23–34.

[40] Paszke A, Gross S, Massa F, Lerer A, Bradbury J, Chanan G, Killeen T, Lin Z, Gimelshein N, Antiga L, Desmaison A, Kopf A, Yang E, DeVito Z, Raison M, Tejani A, Chilamkurthy S, Steiner B, Fang L, Bai J (2019). Chintala S Pytorch: An imperative style, high-performance deep learning library. Adv Neural Inform Process Syst.

[41] Glorot X, Bengio Y. Understanding the difficulty of training deep feedforward neural networks. In: Proceedings of the Thirteenth International Conference on Artificial Intelligence and Statistics, 2010. p. 249–56.

[42] Zajac F, Neptune R, Kautz S (2003). Biomechanics and muscle coordination of human walking: part ii: lessons from dynamical simulations and clinical implications. Gait Posture.

[43] Seene T, Kaasik P (2012). Muscle weakness in the elderly: role of sarcopenia, dynapenia, and possibilities for rehabilitation. Eur Rev Aging Phys Activity.

[44] Dickstein R, Dunsky A, Marcovitz E (2004). Motor imagery for gait rehabilitation in post-stroke hemiparesis. Phys Ther.

[45] Ganguli S, Mukherji P, Bose K (1974). Gait evaluation of unilateral below-knee amputees fitted with patellar-tendon-bearing prostheses. J Indian Medical Assoc.

[46] Ong CF, Geijtenbeek T, Hicks JL, Delp SL (2019). Predicting gait adaptations due to ankle plantarflexor muscle weakness and contracture using physics-based musculoskeletal simulations. PLoS Computat Biol.

[47] Falisse A, Bar-On L, Desloovere K, Jonkers I, De Groote F (2018). A spasticity model based on feedback from muscle force explains muscle activity during passive stretches and gait in children with cerebral palsy. PLoS One.

[48] Plaza A, Hernandez M, Puyuelo G, Garces E, Garcia E (2021). Lower-limb medical and rehabilitation exoskeletons: a review of the current designs. IEEE Rev Biomed Eng..

