# Supplementary material for: Robust walking control of a lower limb rehabilitation exoskeleton coupled with a musculoskeletal model via deep reinforcement learning
Source: J Neuroeng Rehabil. 2023 Mar 19;20:34. doi: 10.1186/s12984-023-01147-2 (PMC10024861; doi:10.1186/s12984-023-01147-2)
Supplement: Supplementary file 1 — Additional file 1. Slides for additional pictures and videos of the lower limb rehabilitation exoskeleton assisting users with different disabilities such as passive muscles (quadriplegic), muscle weakness and hemiplegic conditions. [file 12984_2023_1147_MOESM1_ESM.pptx]

## Slide 1
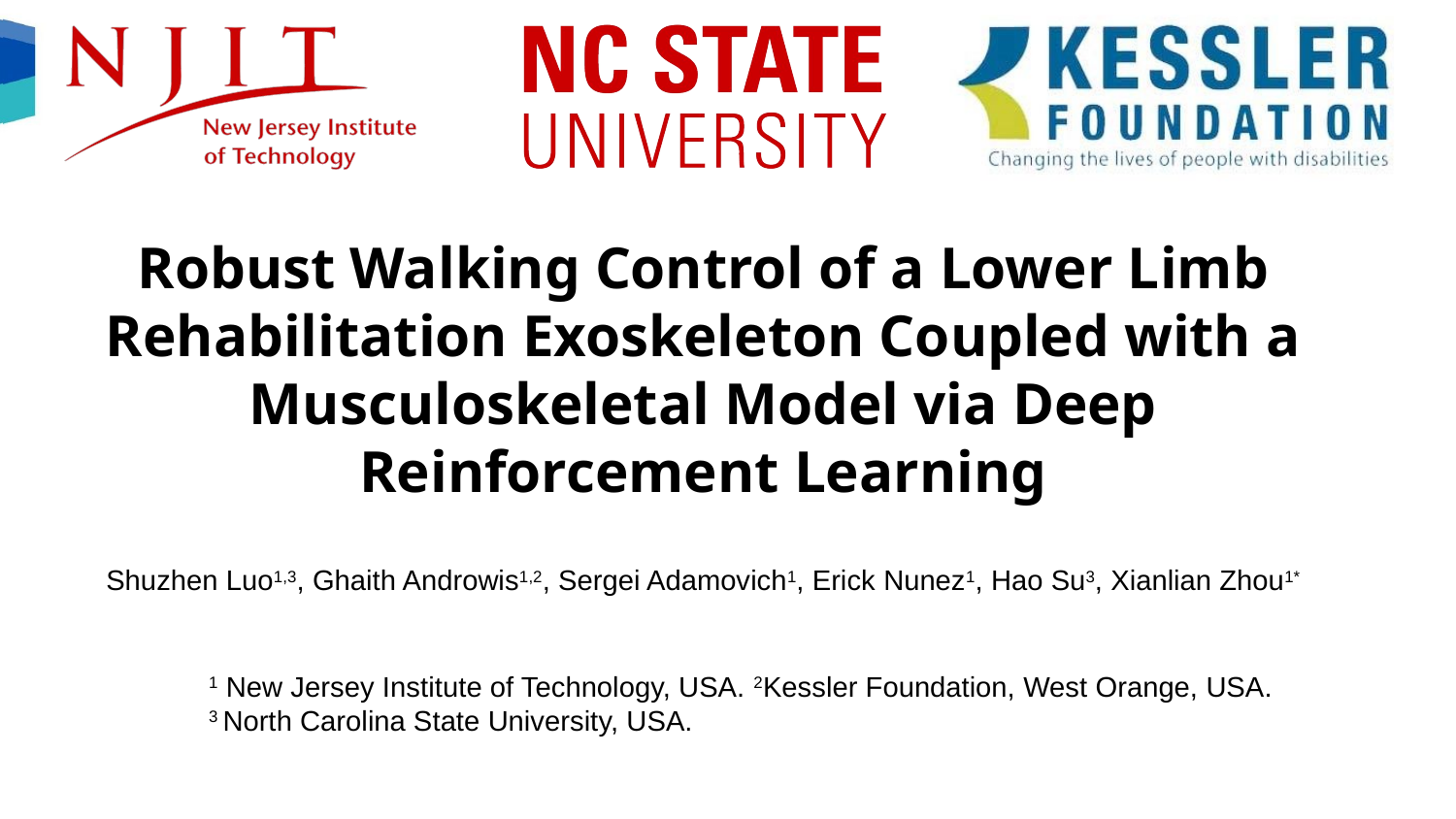

Robust Walking Control of a Lower Limb Rehabilitation Exoskeleton Coupled with a Musculoskeletal Model via Deep Reinforcement Learning
Shuzhen Luo1,3, Ghaith Androwis1,2, Sergei Adamovich1, Erick Nunez1, Hao Su3, Xianlian Zhou1*
1 New Jersey Institute of Technology, USA. 2Kessler Foundation, West Orange, USA.
3 North Carolina State University, USA.

## Slide 2
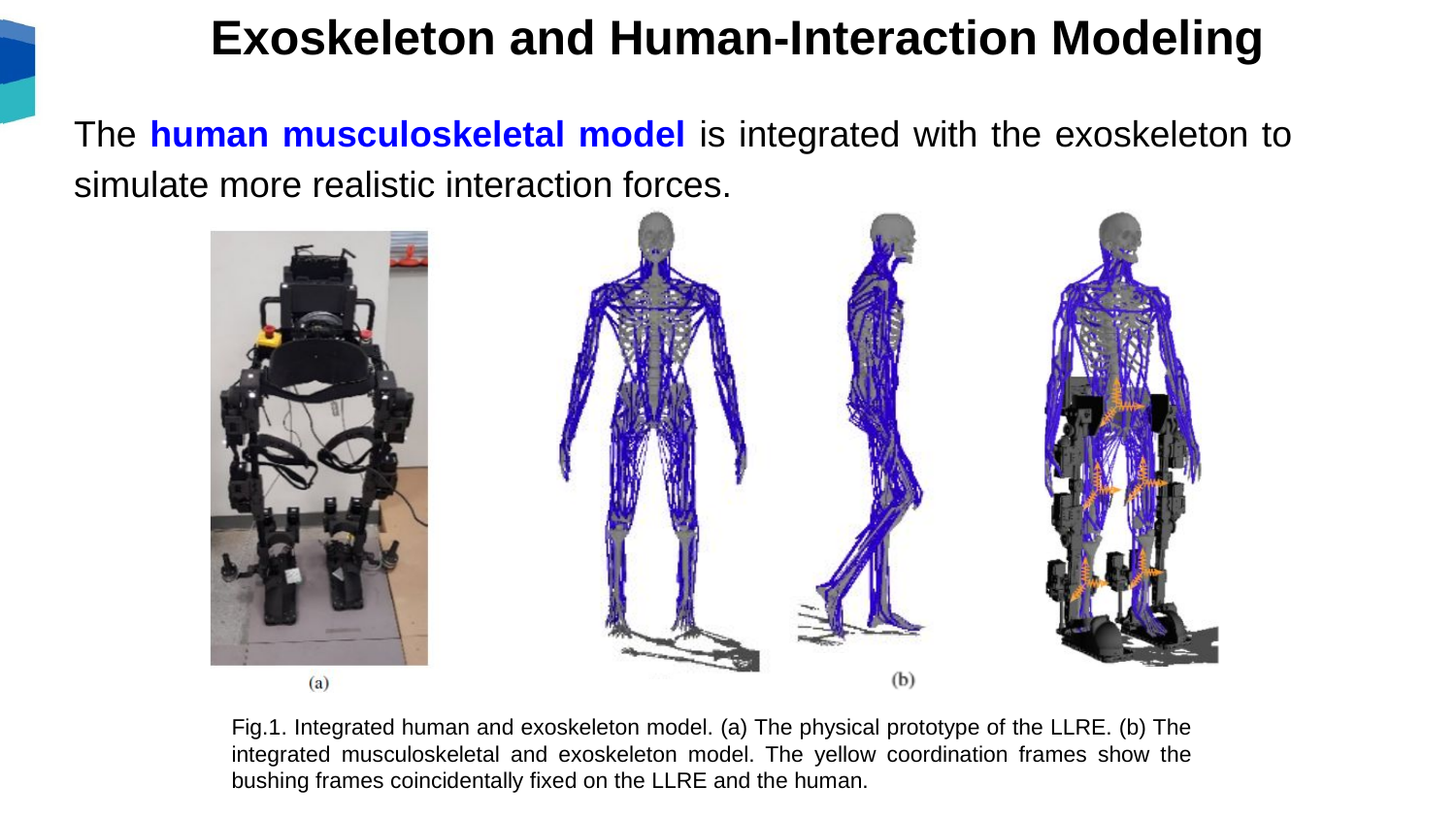

# Exoskeleton and Human-Interaction Modeling
The human musculoskeletal model is integrated with the exoskeleton to simulate more realistic interaction forces.
Fig.1. Integrated human and exoskeleton model. (a) The physical prototype of the LLRE. (b) The integrated musculoskeletal and exoskeleton model. The yellow coordination frames show the bushing frames coincidentally fixed on the LLRE and the human.

## Slide 3
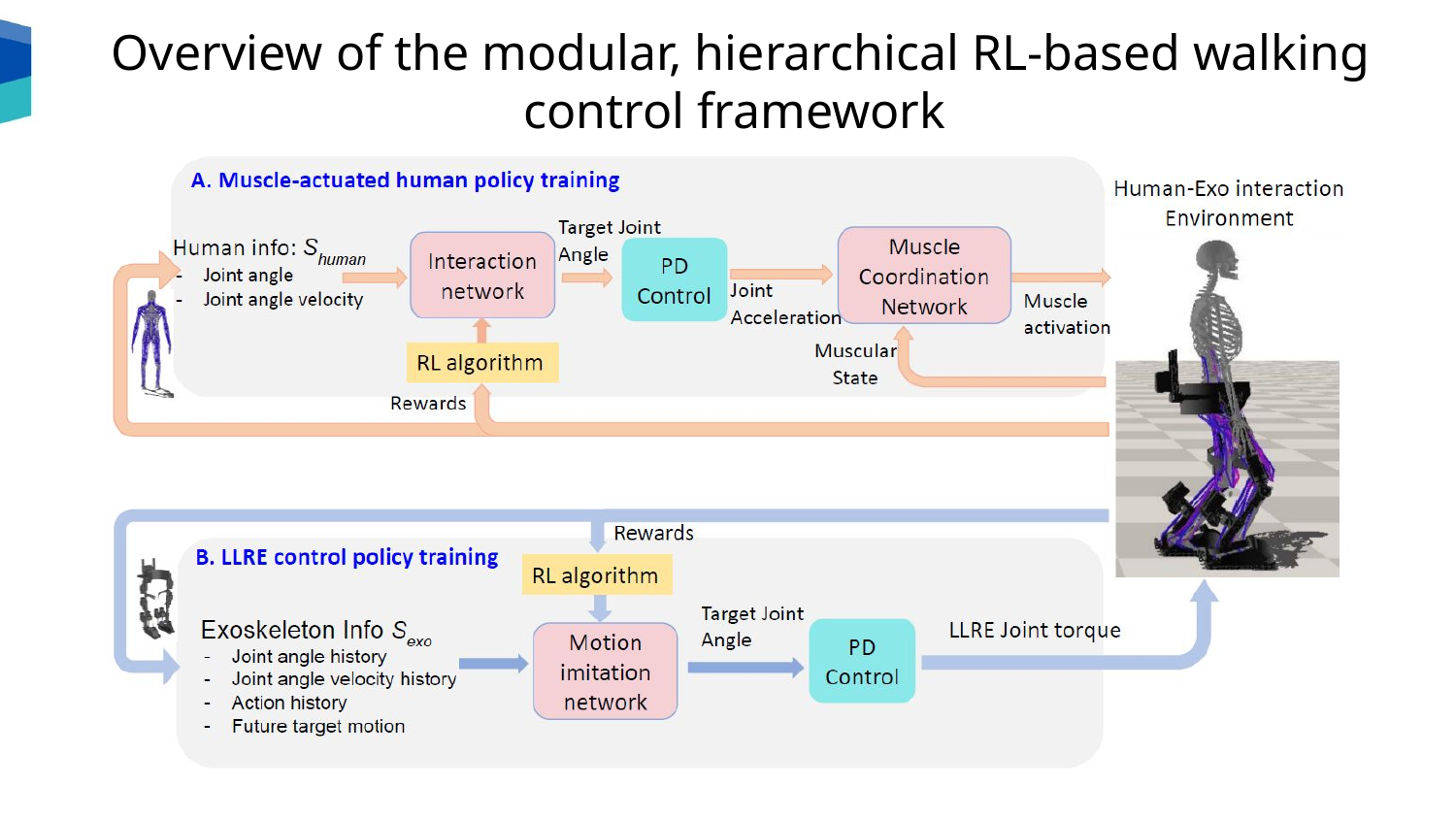

# Overview of the modular, hierarchical RL-based walking control framework

## Slide 4
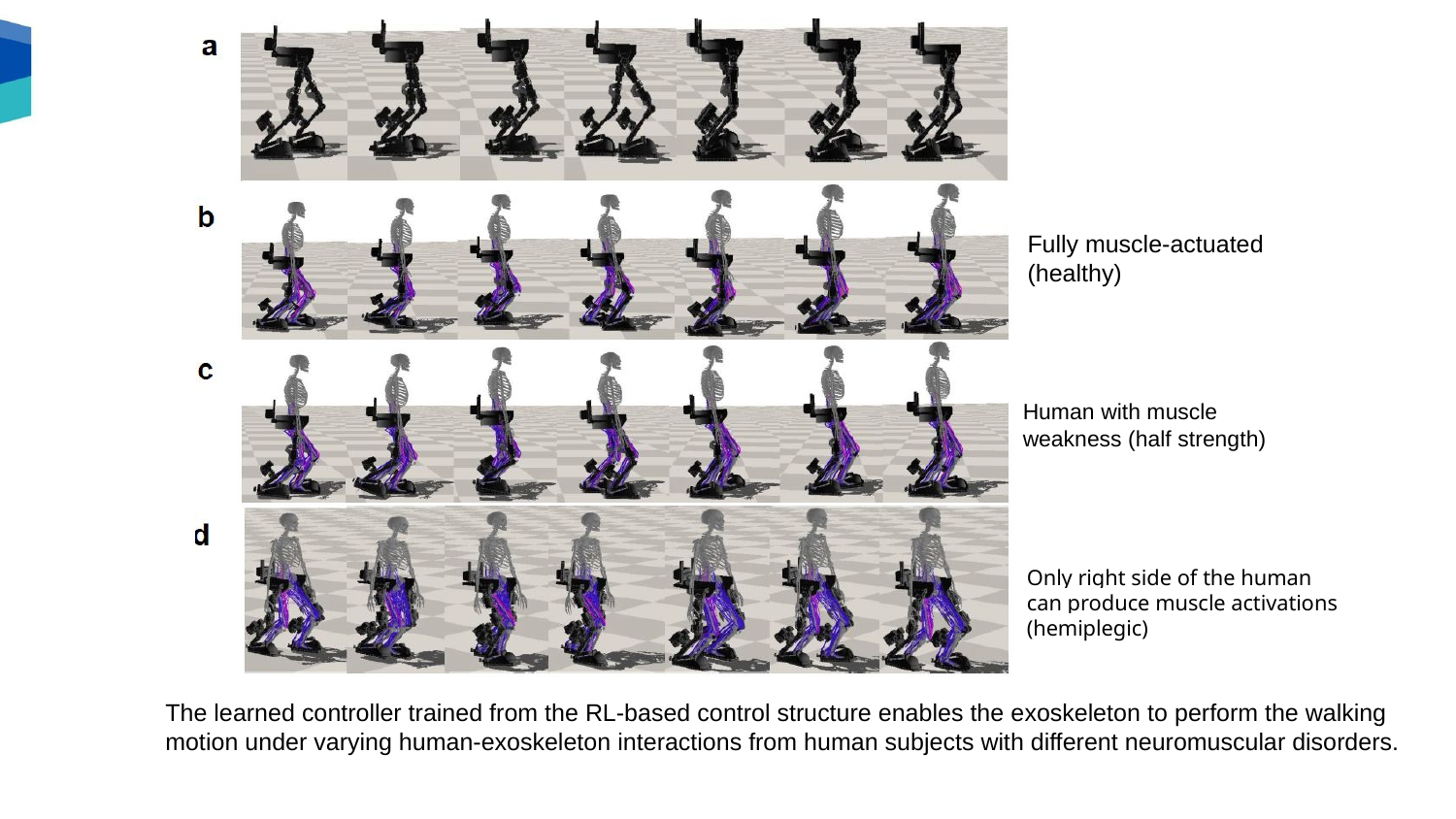

Fully muscle-actuated (healthy)
Human with muscle weakness (half strength)
Only right side of the human can produce muscle activations (hemiplegic)
The learned controller trained from the RL-based control structure enables the exoskeleton to perform the walking motion under varying human-exoskeleton interactions from human subjects with different neuromuscular disorders.

## Slide 5
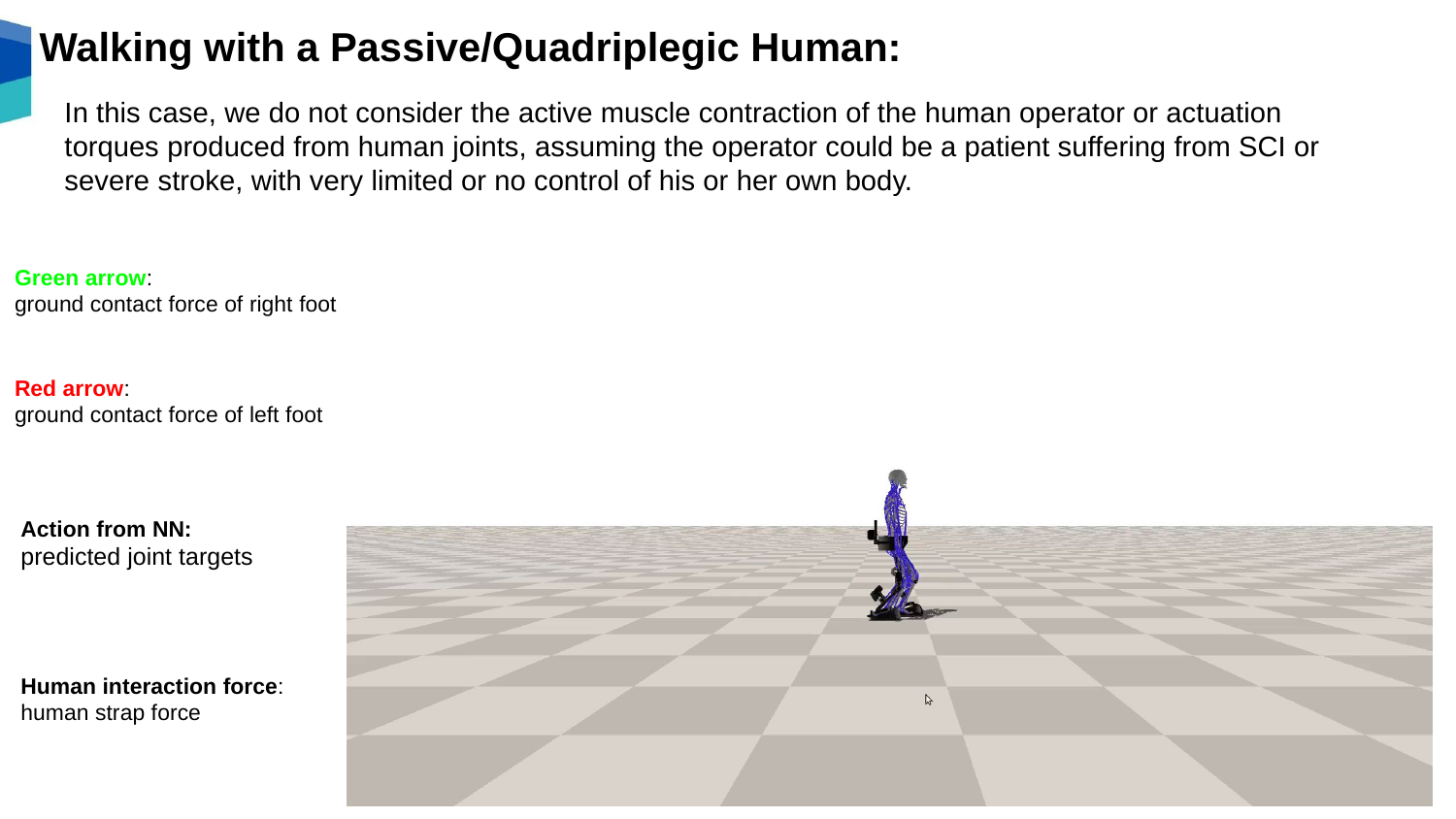

Walking with a Passive/Quadriplegic Human:
In this case, we do not consider the active muscle contraction of the human operator or actuation torques produced from human joints, assuming the operator could be a patient suffering from SCI or severe stroke, with very limited or no control of his or her own body.
Green arrow:
ground contact force of right foot
Red arrow:
ground contact force of left foot
Action from NN:
predicted joint targets
Human interaction force:
human strap force

## Slide 6
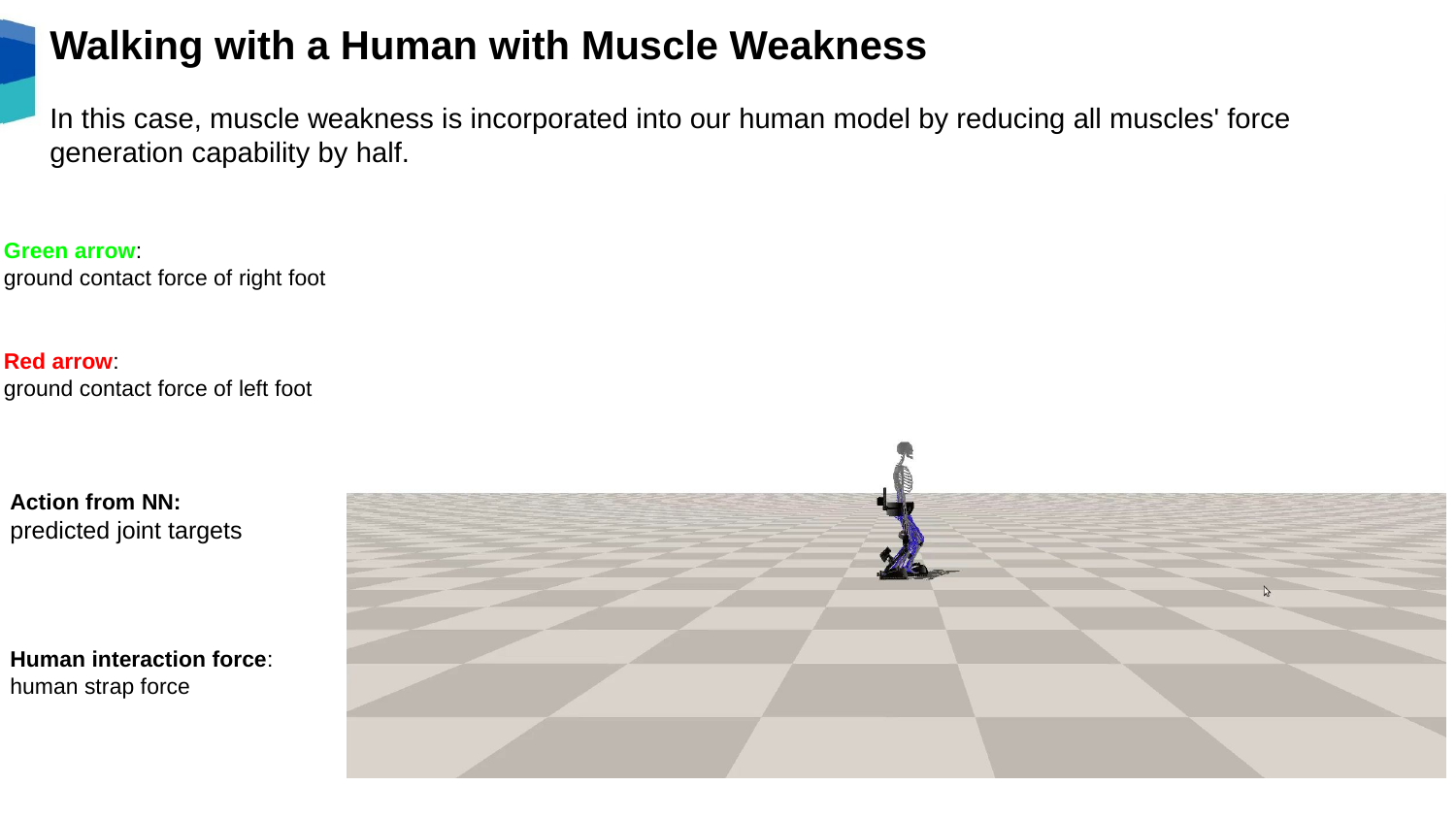

Walking with a Human with Muscle Weakness
In this case, muscle weakness is incorporated into our human model by reducing all muscles' force generation capability by half.
Green arrow:
ground contact force of right foot
Red arrow:
ground contact force of left foot
Action from NN:
predicted joint targets
Human interaction force:
human strap force

## Slide 7
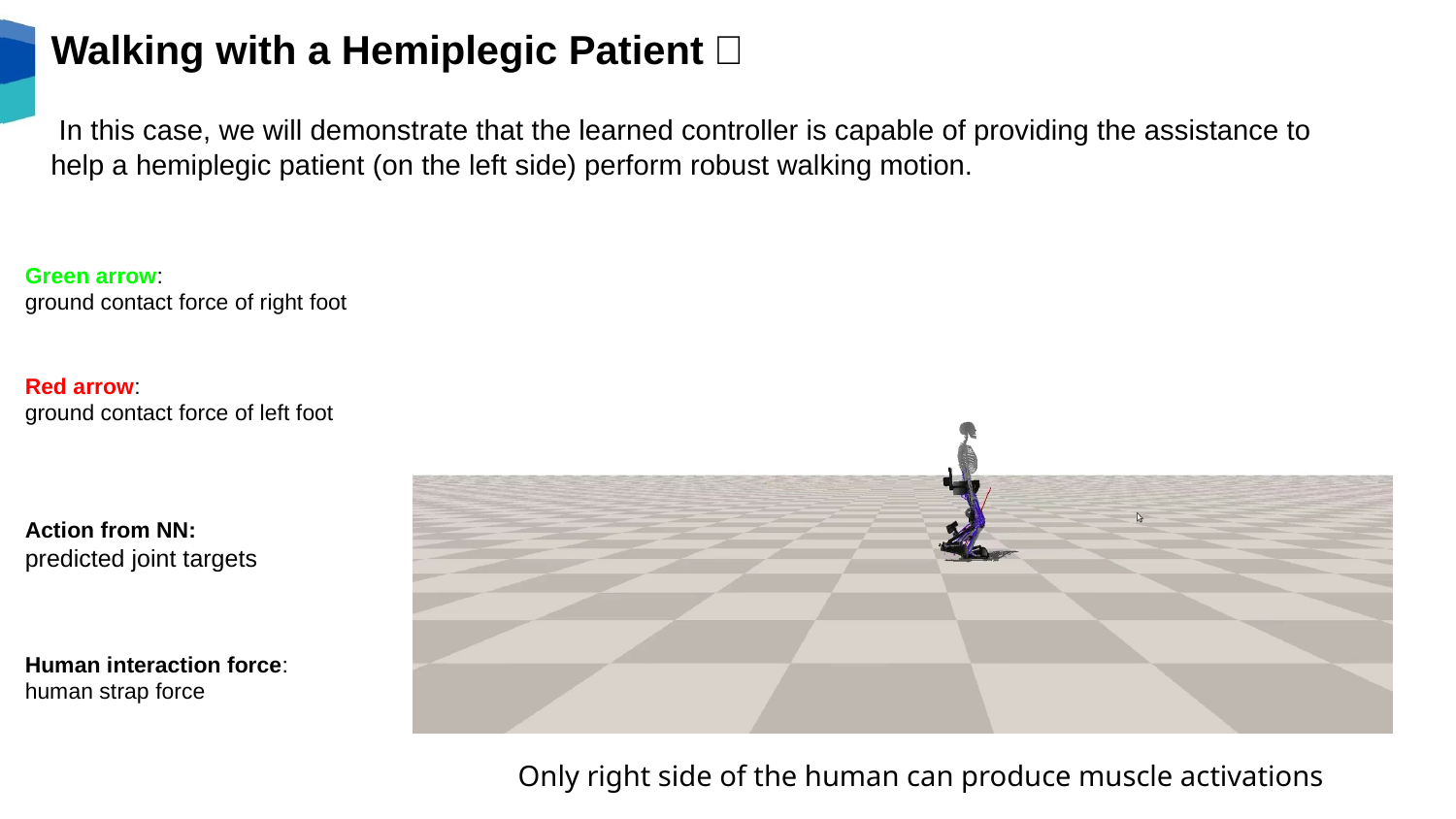

Walking with a Hemiplegic Patient：
 In this case, we will demonstrate that the learned controller is capable of providing the assistance to help a hemiplegic patient (on the left side) perform robust walking motion.
Green arrow:
ground contact force of right foot
Red arrow:
ground contact force of left foot
Action from NN:
predicted joint targets
Human interaction force:
human strap force
Only right side of the human can produce muscle activations
